# Thermo-Mechanical and Thermo-Electric Properties of a Carbon-Based Epoxy Resin: An Experimental, Statistical, and Numerical Investigation

**DOI:** 10.3390/ma17143596

**Published:** 2024-07-21

**Authors:** Giovanni Spinelli, Rosella Guarini, Liberata Guadagno, Luigi Vertuccio, Vittorio Romano

**Affiliations:** 1 Faculty of Transport Sciences and Technologies, Università Telematica Giustino Fortunato, Via Raffaele Delcogliano 12, 82100 Benevento, Italy; 2 Institute of Mechanics, Bulgarian Academy of Sciences, Open Laboratory on Experimental Micro and Nano Mechanics (OLEM), Acad. G. Bonchev Str. Block 4, 1113 Sofia, Bulgaria; rgrosagi@gmail.com; 3 Department of Industrial Engineering, University of Salerno, Via Giovanni Paolo II 132, 84084 Fisciano, Italy; lguadagno@unisa.it (L.G.); vittorioromano2022@gmail.com (V.R.); 4 Department of Engineering, University of Campania “Luigi Vanvitelli”, Via Roma 29, 81031 Aversa, Italy; luigi.vertuccio@unicampania.it

**Keywords:** structural epoxy resin, graphene nanoplatelets, multiphysics simulations, nanocomposites, thermo-electric properties, thermo-mechanical properties

## Abstract

Due to their remarkable intrinsic physical properties, carbon nanotubes (CNTs) can enhance mechanical properties and confer electrical and thermal conductivity to polymers currently being investigated for use in advanced applications based on thermal management. An epoxy resin filled with varying concentrations of CNTs (up to 3 wt%) was produced and experimentally characterized. The electrical percolation curve identified the following two critical filler concentrations: 0.5 wt%, which is near the electrical percolation threshold (EPT) and suitable for exploring mechanical and piezoresistive properties, and 3 wt% for investigating thermo-electric properties due to the Joule effect with applied voltages ranging from 70 V to 200 V. Near the electrical percolation threshold (EPT), the CNT concentration in epoxy composites forms a sparse, sensitive network ideal for deformation sensing due to significant changes in electrical resistance under strain. Above the EPT, a denser CNT network enhances electrical and thermal conductivity, making it suitable for Joule heating applications. Numerical models were developed using multiphysics simulation software. Once the models have been validated with experimental data, as a perfect agreement is found between numerical and experimental results, a simulation study is performed to investigate additional physical properties of the composites. Furthermore, a statistical approach based on the design of experiments (DoE) was employed to examine the influence of certain thermal parameters on the final performance of the materials. The purpose of this research is to promote the use of contemporary statistical and computational techniques alongside experimental methods to enhance understanding of materials science. New materials can be identified through these integrated approaches, or existing ones can be more thoroughly examined.

## 1. Introduction

Composite materials are increasingly attracting significant interest in the research community due to their ability to provide substantial weight savings in production processes, thus broadening the possibilities for designers in every engineering field [1]. These materials are favored because they exhibit remarkable strength-to-weight and stiffness-to-weight ratios, which are much higher than conventional metals [2]. This advantage not only reduces the overall mass of products but also enhances their performance and efficiency. Consequently, the adoption of composite materials is expanding across various industries, including aerospace, automotive, and civil engineering, in which reducing weight without compromising other physical properties is a critical factor [3].

Epoxy polymers are widely utilized as matrices due to their excellent mechanical characteristics and compatibility with various fillers, giving rise to materials with outstanding properties, including high modulus and exceptional chemical resistance [4]. Challenges persist in addressing polymer-based composites’ limited thermal and electrical properties, as they are traditionally acknowledged as insulating materials.

Carbon nanotubes (CNTs) possess remarkable electrical, thermal, and mechanical characteristics, making them highly appealing as fillers in developing nanocomposites to enhance the overall properties of the resulting materials [5]. In fact, technological progress has driven a growing demand for advanced composites possessing exceptional attributes not present in classic materials. Currently, carbon-based nanocomposites with improved electrical and thermal conductivity are increasingly being investigated for potential thermal management applications, such as heating elements, heat exchangers, or heat sinks, driven by a range of potential advantages, including cost, weight, and size reduction of systems, improved chemical resistance, design flexibility, manufacturing process enhancements, and more [6,7].

Uthaman et al. investigated the thermal, mechanical, and water uptake properties of epoxy nanocomposites with surfactant-modified multi-walled carbon nanotubes (MWCNTs) to achieve enhanced mechanical and thermal stability by addressing the common challenge of CNT agglomeration [8]. Li et al. explored the effects of incorporating nanofillers of varying dimensions, including two-dimensional boron nitride and zero-dimensional silica, on epoxy resin’s mechanical and toughness properties [9]. Wu et al. enhanced epoxy resin (Ts) by incorporating thermoplastic resins (polypropylene-PP, polyamide 6-PA6, and polyether-ether-ketone-PEEK) and composites (carbon fiber-PEEK, glass fiber-PA6, and glass fiber-PP). They evaluated mechanical, thermal, and microscopic properties, finding that adding thermoplastic fillers at low ratios (0.5–1.0%) improved tensile, flexural, and shear strength [10].

Zielinski et al. synthesized and studied ionic liquids as latent curing agents for epoxy resins. They demonstrated these compounds’ ability to initiate polymerization and highlighted benefits like extended storage stability and reduced ionic liquid usage [11]. Nguyen et al. presented a novel method to synthesize epoxy networks using phosphonium-based ionic liquids with various anions. They investigated the effect of anion chemistry on polymerization kinetics and the resulting thermal and mechanical properties [12]. The influence of the ionic liquids’ chemical composition on the resulting properties of epoxy networks was also examined, revealing high epoxy group conversion (>90%), high and adjustable glass transition temperatures (>90 °C), and an increased storage modulus [13].

Heat transfer in materials can occur through phonons or a combination of phonons and electrons. In particular, carbon nanotubes or graphene, with their free-moving electrons, offer dual heat conduction, enhancing thermal conductivity compared to other reinforcements that rely solely on phonon transmission. The percolation theory explains the conduction in composites; once the filler concentration hits the percolation threshold, continuous conductive networks form, allowing efficient heat and current flow through lower resistance pathways [14]. Therefore, carbon nanotubes (CNTs) not only improve mechanical characteristics but also impart electrical conductivity to polymers. Consequently, when electrical current or voltage is applied to these conductive materials, Joule heating occurs (self-heating), raising the temperature significantly and potentially causing the heating of the CNT-based composite. Of course, the quality of the morphological arrangement of the filler is essential, as nanoparticles can become defects if the dispersion state is inadequate [15]. Additionally, the particle concentration, aspect ratio, and waviness play critical roles in determining the final performance of the nanocomposites [16]. Carbon nanoparticles usually result in a substantial rise in the nanocomposite’s viscosity, depending on the filler type and loading, which is a crucial factor for the processability of polymers [17,18]. All these factors underscore the significance of continued research endeavors to add knowledge and pursue customized design in the realm of composite materials.

The electric heating behavior of epoxy-based composite films as a function of the applied voltage, as well as the graphene/CNT composition dispersed as fillers, has been investigated by Jeong and An [19]. Isaji et al. [20] presented findings on the self-regulating heating properties of polyethylene films infused with carbon nanotubes. From the viewpoint of potential application as highly efficient flat heaters, the researchers demonstrated that the maximum surface temperature at the equilibrium state of the composite film could be easily managed by adjusting the composite ratio of ultra-high-molecular weight polyethylene (UHMWPE) to low-molecular weight polyethylene (LMWPE). Chatterjee et al. [21] have documented the enhancement of mechanical properties in epoxy composites due to the combined effects of mixed fillers consisting of CNTs and graphene nanoplatelets. Despite the promising results achieved due to their unique properties, nanocomposites present significant challenges stemming from their inherent heterogeneity. Simulation studies are particularly valuable in addressing the gaps in understanding the actual performance of nanocomposites, which often deviates from expected or desired outcomes.

In our previous research [22], a structural epoxy resin filled with varying amounts of carbon nanotubes (up to 3 wt%) was extensively characterized experimentally in terms of mechanical behavior under tensile stress and thermo-electric properties under Joule heating. Starting from preliminary and partial investigations presented in [23], this research marks a significant advancement through its integration of simulations and a statistical approach based on the design of experiments (DoE) to comprehensively analyze the thermo-mechanical and thermo-electric properties of epoxy resin reinforced with selected filler concentrations. For the first time, the authors investigated mechanical properties using FEM simulations by developing a numerical model validated with experimental data. This enabled an in-depth numerical study of mechanical properties such as Von Mises stress and displacement along the dogbone sample, focusing on thermal aspects like strain-induced heating, plastic dissipation energy, and temperature increases at different cross-head speeds, which were previously unexplored. Through DOE, this study also pioneers the investigation of key thermal parameters—such as thermal conductivity, thermal capacity, and heat exchange coefficient—on overall Joule heating. The results provided valuable insights for developing another numerical model, once again validated experimentally, to further explore the thermo-electric properties. These included temperature distribution profiles across the sample and convective heat fluxes due to natural thermal exchange, identifying areas of potential overheating and providing insights into the material’s interaction with its environment. Overall, the findings highlight significant advancements over previous studies, offering a deeper understanding of the complex properties within epoxy nanocomposites and their potential for innovative applications in material science and engineering, particularly in thermal management.

## 2. Materials and Methods

**Figure 1:**
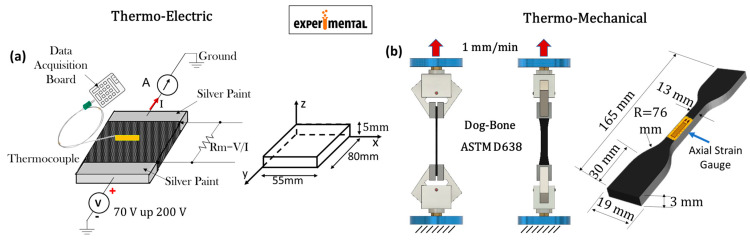
Setup adopted for the experimental thermo-electric and thermo-mechanical measurements, including the geometrical features of the test specimens in (**a**,**b**), respectively.

In the current investigation, nanocomposites for experimental tests were prepared by blending the subsequent components, according to the following procedure detailed in Spinelli et al. [24]: (i) an epoxy matrix, specifically 3,4-epoxycyclohexylmethyl-3’,4’-epoxycyclohexane carboxylate (ECC), serving as the precursor; (ii) a curing agent based on methyl hexahydrophthalic anhydride (MHHPA); and (iii) multiwall carbon nanotubes (commercially known as Arkema Graphistrength^®^ C100) acting as a conductive nanofiller. These constituents were appropriately combined to produce samples with varied CNT concentrations, i.e., [0.3, 0.5, 1.0, 1.5, 2.0, and 3.0] wt%. Notably, the resin employed in this study is classified as a structural epoxy resin due to its prominent mechanical properties. Specifically, the polymerized resin exhibits a storage modulus ranging from 1000 MPa to 2000 MPa over a temperature range of −90 °C to 120 °C. Additionally, it shows a glass transition temperature (Tg) between 150 °C and 250 °C, with a peak value of 205 °C [24]. For the sake of clarity and completeness, more information on the preparation method for the nanocomposites is reported in Section 2, the Materials and Methods of the Supplementary Material file (hereinafter referred to as SM). The essential physical and chemical characteristics of the precursor, hardener agent, filler, and the dimensions of the parallelepiped-shaped test specimens are concisely outlined in Figure S1 of the SI file. Further details can be found in Guadagno et al. [22]. The specimens designated for mechanical characterization were manufactured according to the specifications outlined in the American Society for Testing and Materials (ASTM) D638 standards [25]. The mechanical tests were conducted using a Dual Column Tabletop Testing System (INSTRON, series 5967-INSTRON, Norwood, MA, USA) configured with a cross-head speed of 1 mm/min for both loading and unloading phases. The resulting force was converted into axial stress (σ), and mechanical strain (ε) was calculated as the cross-head displacement of the machine normalized by the gauge length of the specimen under test. Local elongation was monitored using a commercial strain gauge (RS632-180, RS PRO, Corby, UK) affixed to one side of the specimen to prevent slipping phenomena. Changes in its gauge resistance were recorded with a Multimeter 3458A (Agilent, Santa Clara, CA, USA). The current-voltage (I-V) characteristics were measured using a two-probe method on electrical contacts established on the sample surface with silver paint (RS 196-3600, RS PRO, Corby, UK), employing an electrometer, the Keithley 6517A (Keithley Instruments, Cleveland, OH, USA), acting as both the power supply and ammeter. This measurement approach has been deemed suitable in the literature for electromechanical characterization [26,27]. The contact resistance can be disregarded, as it is lower than the overall electrical resistance of the specimens (several kΩ). To experimentally investigate the Joule heating effect, silver paint (brand name: RS 196-3600, RS PRO, Corby, UK) was deposited onto the short sides of the test specimens to establish ohmic contacts between the power supply EA-PSI 8360-10T (Elektro-Automatik, 0–360 V, 0–10 A, 1 kW max, Viersen, Germany) and the HP34401A ammeter (min current 0.1 μA, Hewlett-Packard Company, Loveland, Colorado, USA) using the 2-probe method. The surface temperature increase over time was then monitored under various applied voltage values (specifically 70 V, 80 V, and 90 V) using a T-type thermocouple positioned at the center of the upper surface of the sample. The temperature values were automatically recorded using a data acquisition board (Data Logger TC-08 supplied by Pico Technology), with the assistance of the PicoLog software (version 6.2.5). The schematic diagrams of the experimental setups used to investigate the thermo-electric phenomenon due to the Joule effect and the thermo-mechanical properties are illustrated in Figure 1a,b. The geometric specifications of the corresponding samples are also provided.

For simulation studies on mechanical properties and strain-induced heating, as well as for thermo-electric aspects related to the Joule effect, the commercial software COMSOL Multiphysics^®^ (version 6.1) based on the finite element method (FEM) was adopted. This allowed the authors to perform a numerical/experimental comparison of the results with the aim of validating the numerical models for reliable use in further physical investigations on the analyzed samples.

**Figure 2:**
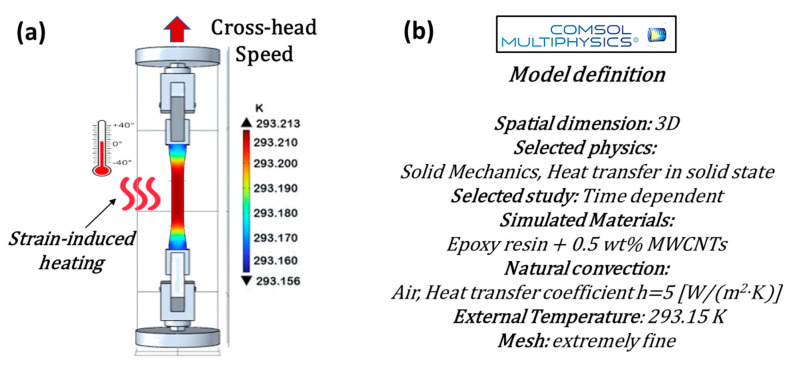
(**a**) Thermo-mechanical case study addressed in the present study; (**b**) main model definitions for the numerical investigation adopted in COMSOL Multiphysics^®^.

**Figure 3:**
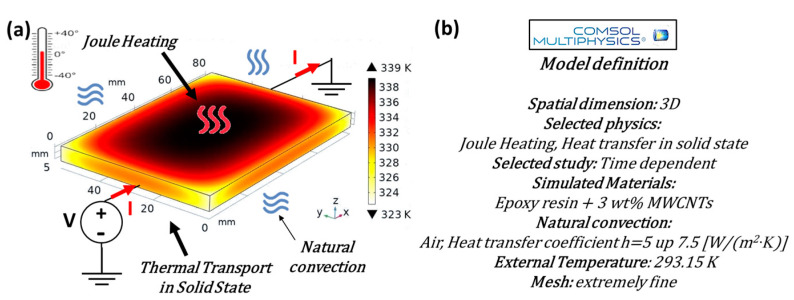
(**a**) Thermo-electric case study addressed in the present study; (**b**) main model definitions for the numerical investigation adopted in COMSOL Multiphysics^®^.

Figure 2 and Figure 3, respectively, present the schematic representations and essential model definitions for the thermo-mechanical and thermo-electric case studies investigated in the current work, in parts (a) and (b) of each figure.

In brief, in both cases, the models are defined in a three-dimensional (3D) space, allowing for a comprehensive analysis of the physical properties of the samples evaluated with a time-dependent study to capture the dynamic response of the materials.

Additional information regarding the physics adopted for the simulations and specific values used during parameter setup, as well as the thermal balance equation (Equation (S1)) and the boundary conditions for its unique resolution (Table S1), are detailed in the Supplementary Materials (SM).

The design of experiments (DoE) methodology falls within the realm of applied statistics and analyzes cause-and-effect relationships. This approach identifies how different factors influence a process or product and, consequently, its output or performance function (P.F.).

A concise yet detailed description of this technique, including its schematic representation (Figure S2), is provided in the SM.

In this study, the design of experiments (DoE) is employed to evaluate the impact of three thermal variables on the temperature distribution of the nanocomposites. These variables are the heat transfer coefficient in still air (*h*, natural convection), thermal conductivity (*λ*), and thermal capacity (*C*). In particular, the surface temperature is evaluated at time t = 240 s (T-240s) during the transient phase and at t = 3600 s (T-3600s), which corresponds to the steady-state temperature (plateau value).

Therefore, both these temperatures serve as the focal performance metrics in this DoE study. To facilitate this analysis, a dedicated Matlab^®^ (version 2015) routine has been developed. For a successful application of DoE, it is essential to establish an appropriate discretization level for the input variables. Initially, a uniformly distributed set of parameter values is recommended, with the possibility of incorporating additional intermediate points later to enhance model refinement [28]. In our study, the input variable vector $\bar{x}$ has been defined as follows:

(1) $$\bar{x}=(h,\lambda ,C)\epsilon R^{3}$$

where a discretization on 3 levels is applied to the selected input factors within the following intervals: [5, 7.5], [0.100, 0.150] and [9.21, 14.5], for *h*, λ, and *C*, respectively.

In more detail, the considered values are as follows:

(2) $$h_{1}=5,{\;h}_{2}=6.25,h_{3}=7.5$$

(3) $$\lambda_{1}=0.100,{\;\lambda }_{2}=0.125,\lambda_{3}=0.150$$

(4) $$C_{1}=9.21,C_{2}=11.8,C_{3}=14.5$$

Consequently, the compact D that describes the variable space is mathematically expressed as follows:

(5) $$D=h\times \lambda \times C\subset R^{3}$$

whereas the *P.F*. is evaluated for each ordered term $\left(x_{1},x_{2},x_{3}\right)$ of the input variable vector, as follows:

(6) $$\left(x_{1},x_{2},x_{3}\right)=(h,\;\lambda ,C)\epsilon D.$$

## 3. Results

The next subsections present the comprehensive results of our investigation into the thermo-mechanical and thermo-electric properties of composite materials. Both experimental data and theoretical models are analyzed to provide a thorough understanding of the behavior of these materials under various conditions. A comparative analysis between the experimental results and theoretical predictions was performed to validate the accuracy and reliability of the models used.

### 3.1. Experimental Results on Percolation Curve

**Figure 4:**
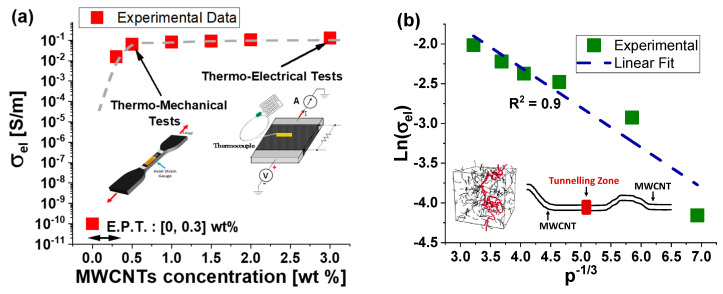
Electrical conductivity as a function of filler concentrations in (**a**); tunneling verification in nanocomposite systems in (**b**).

Percolation refers to the process by which at least a continuous pathway of conductive filler particles forms within an insulating matrix as the filler concentration increases. Individual filler particles are isolated within the matrix at low filler concentrations, resulting in poor electrical conductivity. Otherwise, as the filler concentration (*p*) exceeds a critical threshold (the so-called percolation threshold, hereafter, p_c_), the filler particles begin to connect, forming a percolating network that allows for efficient electron transport and a significant increase in electrical conductivity (σ_el_). Experimental determination of the percolation threshold and the overall percolation curve involves measuring the electrical conductivity (see Figure 4a) of nanocomposite samples prepared with varying filler amounts. As expected, the relationship between the filler concentration and the electrical conductivity of the nanocomposite material can be mathematically described using a power law equation that takes the following form:

(7) $${\sigma_{el}}_{\;}={\sigma_{0}\cdot \left(p-p_{c}\right)}^{t}\;\;\;\;for\;\;p>p_{c}.$$

Here, σ_0_ represents a pre-factor related to the intrinsic electrical conductivity of filler, whereas t is the critical exponent, i.e., a dimensionless parameter that characterizes the steepness of the conductivity increase as the filler concentration approaches and exceeds the percolation threshold.

It can be observed that thanks to the good dispersion of carbon nanotubes within the epoxy matrix, the electrical percolation threshold is reached at low filler concentrations in the range of [0–0.3] wt%. An electrical conductivity value of 15.65 × 10^−3^ S/m is measured for a concentration of 0.3 wt% of MWCNTs, which is significantly higher than that exhibited by the pure resin (order of 10^−10^ S/m). Further increasing the concentration of MWCNTs results in a slight increase in electrical conductivity, which reaches a maximum value of 0.13 S/m at the highest concentration investigated in this study (3 wt%). In nanocomposite materials, tunneling is the primary mechanism for electric transport, in which electrons traverse potential barriers through quantum mechanical tunneling rather than conventional conduction pathways. The validation of the tunneling mechanism in nanocomposite materials can be achieved by analyzing (see Figure 4b) the natural logarithm of conductivity as a function of concentration at p^−1/3^.

The investigation of the percolation phenomenon provides valuable insights into the electrical and other behaviors of nanocomposite materials, guiding their design and optimization for various engineering applications. Based on the discussed results, composites with a filler concentration of 0.5 wt% were selected for mechanical tests, while those with a concentration of 3 wt% were chosen for thermo-electrical tests.

This is because a concentration near the percolation threshold (i.e., 0.5 wt%) is ideal for exploring the strain sensor properties of composites. At this concentration, the percolation network is just formed, and it is more sensitive to imperceptible variations due to applied strain, unlike the well-established network achieved at higher filler concentrations. In nanocomposite materials, like those incorporating carbon nanotubes (CNTs), the concentration of CNTs is crucial for their properties and performance in various tests, including axial strain and piezoresistive tests. The concentration of CNTs at 0.5 wt% is just above the EPT. Hence, the conductive network is newly formed and relatively sparse. This makes the network more sensitive to deformations, such as those caused by axial strain. As strain is applied, the distances between CNTs change, causing significant changes in electrical resistance. This high sensitivity is beneficial for detecting small strains accurately. The morphological network is denser and less affected by deformation at higher concentrations. Moreover, such a concentration of 0.5 wt% just above the EPT is optimal for sensitivity and ensures adequate overall electrical conductivity. Adequate conductivity is essential to minimize measurement errors during electrical evaluations. If the conductivity is too low, the resistance changes due to strain might be difficult to distinguish from noise or other sources of error. Thus, selecting this concentration strikes a balance between sensitivity and reliable conductivity, making it ideal for such tests. Differently, a higher concentration (3 wt%) is recommended for analyzing the thermo-electric properties of the composites due to the Joule heating effect, which requires adequate conductivity values. In fact, at the highest concentration above the EPT, the CNT network forms a continuous conductive path throughout the material. This enhances electrical conductivity significantly compared to concentrations below or around the threshold, at which conductivity may be sporadic or insufficient for practical applications. Higher conductivity means more efficient conversion of electrical energy into heat (Joule heating). This efficiency is crucial in applications requiring precise control and heating uniformity, such as electrical heaters or heat exchangers. Moreover, carbon nanotubes are known for their excellent thermal stability. At higher concentrations, the network of CNTs can dissipate heat effectively without degradation or hot spots, ensuring uniform heating and prolonged operational stability, as well as the durability and reliability of the material in applications in which temperature fluctuations or mechanical stresses could compromise performance. The results of these investigations are presented in the next subsections.

### 3.2. Mechanical and Piezoresistive Properties: Experimental Results

**Figure 5:**
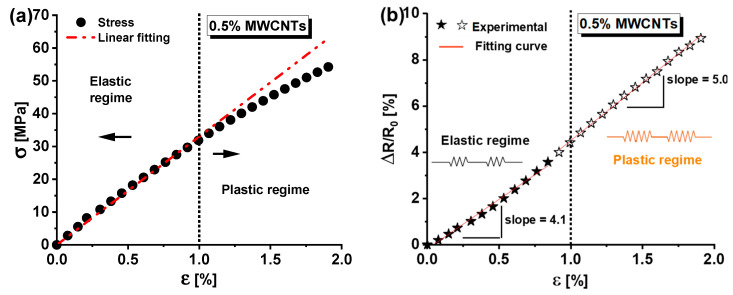
(**a**) Mechanical behavior and (**b**) resistance change ratio versus axial strain for samples reinforced with 0.5 wt% of MWCNTs.

The mechanical response of nanocomposite materials under axial tensile load is a crucial aspect of their characterization. Figure 5a depicts the mechanical behavior due to axial tensile stress of the specimen containing 0.5 wt% of MWCNTs, which was observed until failure that occurred at a strain value of approximately ε = 2%.

For clarity, the strain ε is defined as the change (expressed in %) in length Δl divided by the initial length L_0_, i.e., ε =Δl/L_0_. Moreover, a linear response is observed up to a strain value of approximately ε = 1%. Beyond this point, a deviation from the linear response indicates the onset of plastic deformation and permanent damage.

This transition threshold is critical for the material’s structural design; below it, the material maintains its structural integrity (elastic region), whereas, beyond this threshold, the nanocomposite undergoes non-recoverable deformation (plastic regime) due to irreversible changes in the morphology of the resin network.

Similar considerations can be drawn by observing (see Figure 5b) the change in electrical resistance (ΔR/R_0_, in percentage), mainly due to the percolation network, as it varies with mechanical strain (ε). Here, R_0_ represents the electrical resistance of the unloaded specimen (i.e., ε = 0), and ΔR = R − R_0_ represents the change in resistance due to the applied load. In particular, within the material’s elastic region (i.e., ε < 1%), an initial linear relationship between ΔR/R_0_ and applied strain ε was observed, with a fitting curve of the experimental data characterized by a slope of 4.1. As the strain increases, an elevated slope of five is revealed for this curve. This change is most likely attributed to plastic deformation as the material exceeds its elastic limit.

These variations are electrically noticeable because a nanocomposite with a concentration (0.5 wt%) of filler near the percolation threshold, thus having a morphological structure highly sensitive to small deformations, was considered. Finally, it is worth noting that applying mechanical stress to a material generates heat due to the conversion of mechanical energy into thermal energy. In fact, when a material is subjected to mechanical stress, the internal structure of the material is deformed, resulting in frictional forces between its molecules. This friction generates heat, causing an increase in temperature within the material. Such aspects will be numerically investigated in the next sub-section.

### 3.3. Thermo-Mechanical Properties: Simulation Results

#### 3.3.1. Validation Model

**Figure 6:**
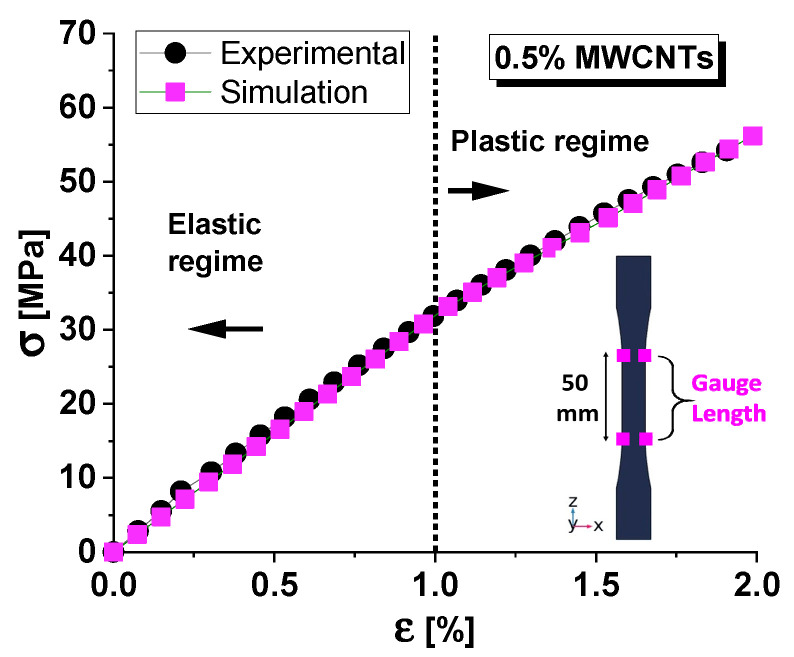
Comparison between experimental and simulated data for model validation.

As the initial step in validating the numerical model for mechanical investigations, Figure 6 compares experimental and simulation results for the stress–strain behavior of the selected nanocomposite material. The inset illustrates the 50 mm gauge length used for stress evaluation, as the standard requires. In fact, the load vs. strain curve for a composite material with a dogbone structure can vary significantly at different points along the length of the sample. This variation is due to the different distribution of stresses and strains in the sample, which is influenced by its variable geometry [29].

It is interesting to note that there is a strong correlation between the experimental and simulation results. The data points from both methods closely follow the same trend, indicating that the simulation model is well-calibrated and accurately predicts the material’s behavior. The transition from elastic to plastic regime (yield point) is clearly marked at around 1% strain. Both experimental and simulation results align well at this transition point, further validating the accuracy of the simulation. Beyond this point, the curve continues to rise but at a reduced slope, indicating that plastic deformation occurs, and the material does not return to its original shape when the load is removed.

#### 3.3.2. Simulation Results on Z-Axis Displacement and Von Mises Stress

**Figure 7:**
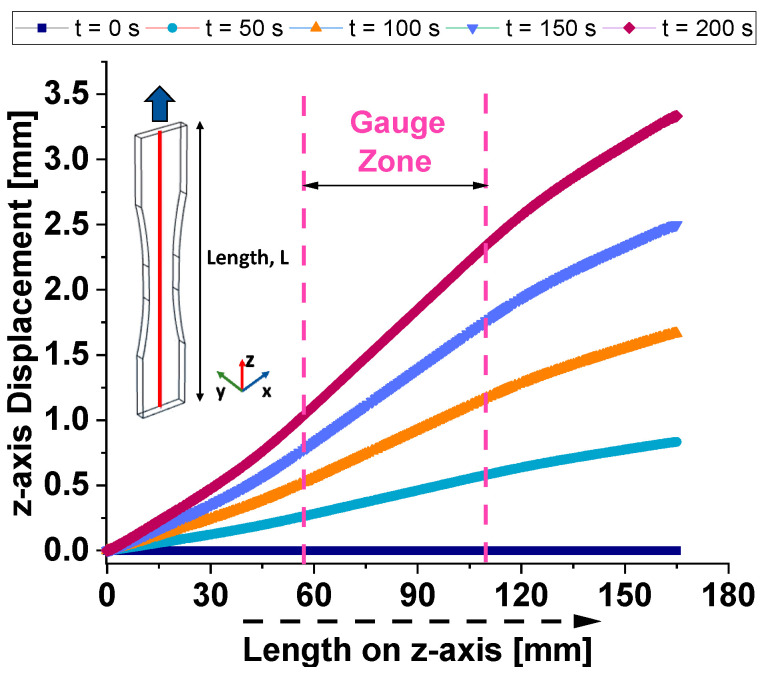
Z-axis displacement versus the dogbone length along the z-symmetry axis.

When a tensile force is applied, the material experiences elongation along the z-axis, typically the applied load’s direction. Figure 7 illustrates the z-axis displacement versus the length along the z-axis of symmetry (see the red line of the inset) for the considered dogbone-shaped sample at different time instants (t = 0, 50, 100, 150, and 200 s).

The initial displacement is zero, serving as a baseline for subsequent measurements. It is possible to note that the z-axis displacement increases with time, indicating progressive deformation of the sample and, therefore, the time-dependent strain behavior of the composite material. At each given time, the displacement is larger as we move toward the higher z-axis length, suggesting a non-uniform deformation along the length of the sample. The gauge zone, marked between approximately 60 mm and 110 mm along the z-axis, is the region of interest in which deformation measurements are most relevant. The displacement within this zone is notably higher, indicating that this region experiences the most significant strain during the test. At t = 50 s, the related curve shows a small but noticeable increase in displacement, suggesting the beginning of strain in the material. The curve at t = 100 s indicates further displacement, with a more pronounced increase than the previous times. Strain is more evident, especially in the gauge zone. A significant increase in displacement, reflecting accelerated deformation over time, is observable at t = 150 s. The gauge zone continues to display substantial strain, with displacement values considerably higher than at 100 s. Finally, the last curve (at t = 200 s) shows the maximum observed displacement, indicating continuous strain accumulation. The deformation is most pronounced within the gauge zone, suggesting that the material is experiencing progressive failure or significant plastic deformation.

**Figure 8:**
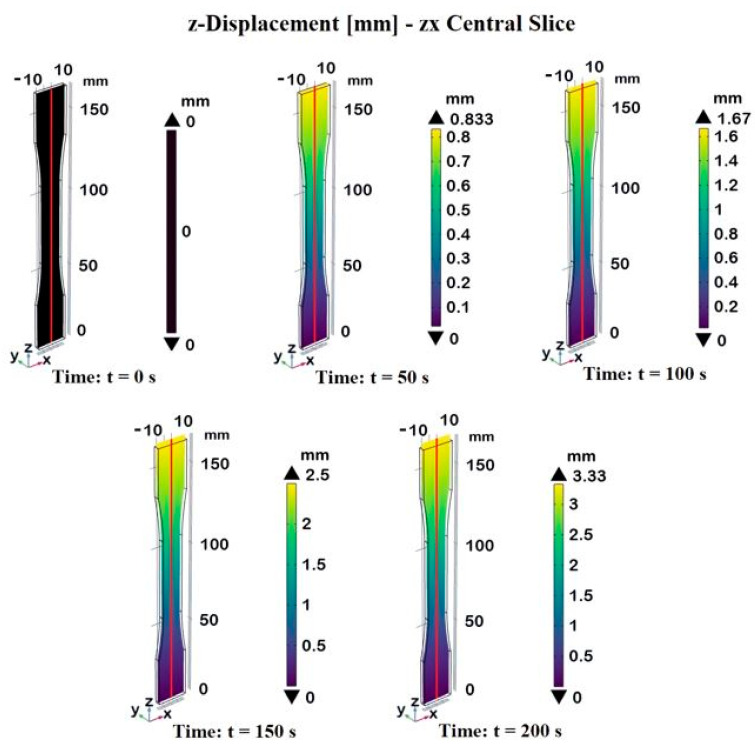
3D z-axis displacement versus the dogbone length on the central slice of the zx-plane passing through the axis of symmetry in the z-direction.

Figure 8 shows the 3D displacement profiles of the dogbone sample made of composite materials at different times, as follows: t = 0 s, t = 50 s, t = 100 s, t = 150 s, and t = 200 s. The displacement is measured on the central slice of the zx-plane passing through the axis of symmetry in the z-direction. The color bar indicates the magnitude of displacement in millimeters (mm).

At the initial state (t = 0 s), there is no load applied to the sample, so the displacement is uniformly zero across the entire dogbone length, as indicated by the black color. As time progresses, the dogbone sample starts to experience displacement. The reported plots illustrate a progressive increase in displacement over time, with the maximum displacement reaching 3.33 mm at 200 s. The color bar effectively highlights the differences in displacement magnitude, providing a clear visual representation of the deformation behavior of the composite material under tensile stress over time.

**Figure 9:**
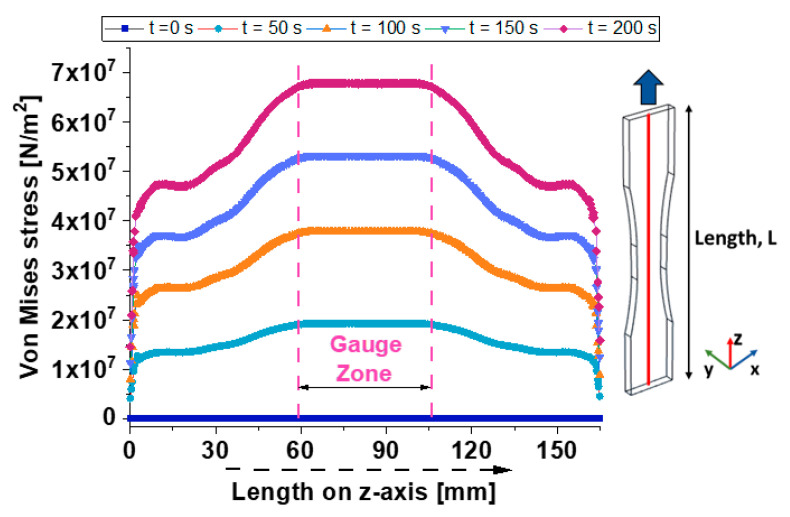
Von Mises stress profiles versus the dogbone length along the z-axis of symmetry.

Figure 9 illustrates the distribution of Von Mises stress recorded on the total length (165 mm) of dogbone alongside the axis of symmetry in the z-direction (see the schematic on the right part) at the selected time instants (t = 0, 50, 100, 150, and 200 s).

From the analysis of these plots, it is worth noting that the Von Mises stress increases with time, indicating progressive stress accumulation in the material.

For each time, the stress profile shows a peak within the gauge zone, which is the region most subjected to stress concentration. This indicates that it is the critical region for assessing the material’s stress response.

Of course, at time t = 0, the initial stress distribution shows a baseline with null stress, as expected at the start of the test.

The curve at time t = 50 s shows an increase in Von Mises stress, with a noticeable peak forming in the gauge zone. This indicates the beginning of significant stress development within the material that decreases moving away from this particular area. At the time t = 100 s, the representative curve shows a further increase in stress, with the peak in the gauge zone becoming more pronounced.

Stress levels outside the gauge zone also rise but remain lower compared to the peak region. The curve at time t = 150 s presents a substantial increase in stress, especially within the gauge zone. The stress distribution is more symmetric, with the highest stress values of about 5 × 10^7^ N/m^2^ reached in the mid-length of the sample.

The last curve relating to the time t = 200 s indicates the maximum observed stress, with the peak stress in the gauge zone exceeding 5 × 10^7^ N/m^2^.

This suggests significant stress accumulation, likely approaching the material’s failure threshold.

In all cases, the stress distribution suggests that the composite material undergoes significant stress accumulation over time, with the highest stress concentrations occurring in the central gauge zone. This behavior is typical for materials subjected to tensile loading, where the narrowest section experiences the greatest stress.

**Figure 10:**
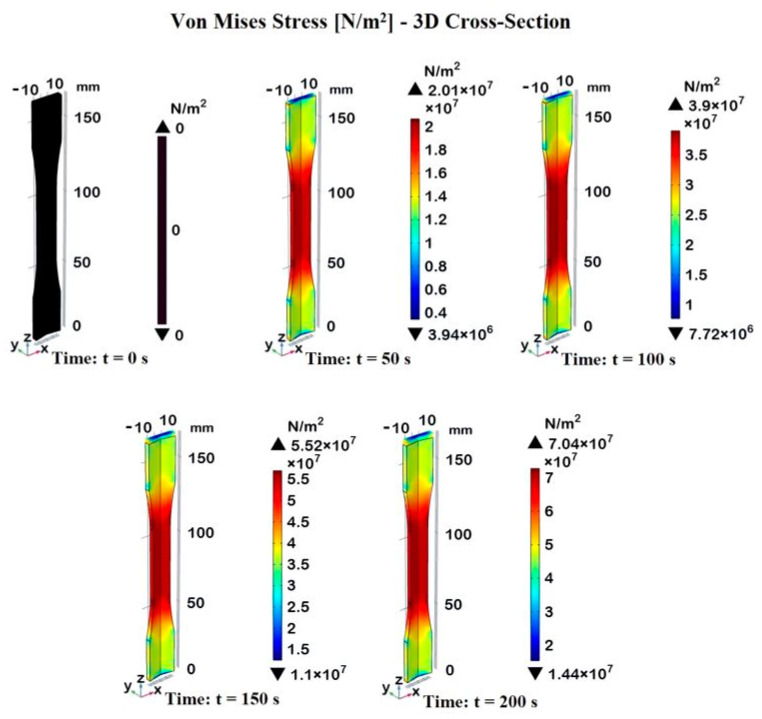
Von Mises stress profiles (3D cross-sections) versus the dogbone’s length. The profile along the symmetry axis in the z-direction is clearly visible.

Figure 10 illustrates, with a 3D cross-section, the Von Mises stress distribution in the dogbone sample at the usual time instants.

The color bar represents the stress values in units of 10^7^ N/m^2^, providing a clear visualization of the stress distribution at each time instant.

At t = 0 s, there is no applied load and, consequently, no stress is observed in the sample. The entire sample is uniformly black, indicating a null Von Mises stress. As the tensile load is applied and time progresses, the stress in the sample increases, with the maximum stress concentrated in the gauge section and a maximum peak (7.04 × 10^7^ N/m^2^) reached at time t = 200 s.

Regardless of the time instant, the central portion of the specimen experiences the highest stress, shown in the red region.

This is typical in tensile tests, in which the narrower section (gauge zone) of the specimen bears the maximum load.

The choice to report a 3D cross-section view effectively allows for the observation of the distribution and evolution of Von Mises stress at various points in the specimen and at different time intervals, highlighting the regions of highest stress concentration and their temporal progression

#### 3.3.3. Strain-Induced Heating and Plastic Energy Dissipation Density

The relationship between heat-generated and applied strain in a material is often described by the concept of “strain-induced heating”.

When a composite material is subjected to mechanical loading, part of the mechanical work performed on the material is converted into heat.

This phenomenon is particularly evident in materials exhibiting viscoelastic behavior or undergoing plastic deformation.

**Figure 11:**
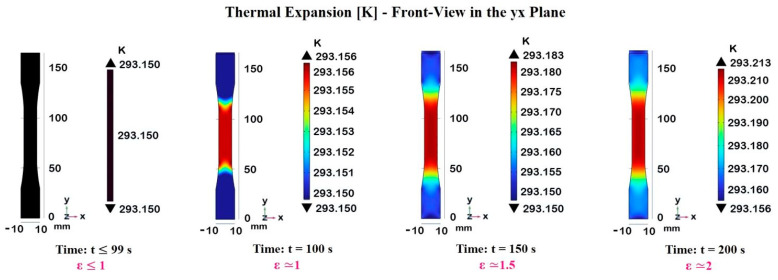
Temperature distribution in the dogbone sample at different time instants corresponding to different strain levels.

In our case study, it is possible to numerically investigate this, even though the effect is not large due to the low thermal coefficient exhibited by the epoxy resin (about 60× 10^−6^/K) and the limited level of plastic strain investigated, according to the experimental mechanical test (from 1% up to 2%). Figure 11 illustrates the temperature increase in our dogbone-shaped composite material sample under tensile strain at different time intervals, as follows: t = 0 s up to 99 s, corresponding to strain levels in the elastic region, t = 100 s, at which ε is about 1% (yield point), t = 150 s (ε ≃ 1.5%), and t = 200 s (ε ≃ 2%), conforming to strain levels in the plastic region.

The temperature distributions are graphically displayed for each case, and their values are provided by the color bar. Of course, up to 99 s, the sample remains in the linear elastic region. There is no significant temperature increase, as the uniform black color indicates a base temperature of 293.150 K. The sample has not yet entered the plastic deformation zone, and no thermal heating is observed.

As the strain level increases above the yield point entering the plastic deformation zone, the temperature within the sample also starts to increase. This is because, in this region, the mechanical work is no longer stored as elastic potential energy; instead, some of it is converted into heat due to internal friction.

At 200 s, the sample is well into the plastic deformation zone, and the temperature increase is evident.

The heat distribution becomes more concentrated and localized in the gauge region subjected to higher strain levels, indicating potential hotspots that could affect the material’s mechanical properties and performance. The temperature in the central gauge section reaches a maximum of approximately 293.213 K.

The cross-head speed $\dot{ʋ}$ impacts the temperature distribution within a composite material during deformation. Higher strain rates generally lead to increased heat generation due to more intense mechanical work on the material in a shorter period.

This is evident from the progressively higher maximum temperatures as the crosshead speed increases.

**Figure 12:**
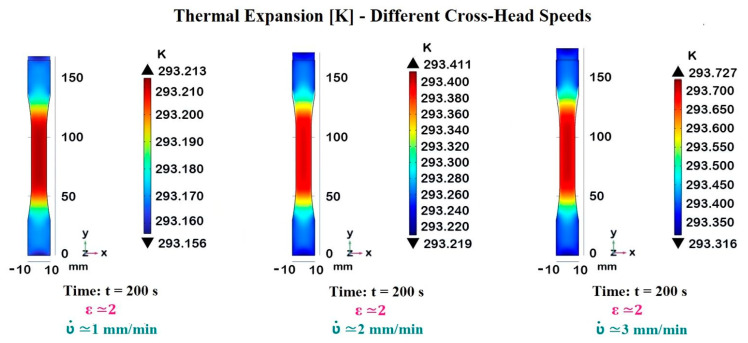
Thermal expansion in the dogbone sample for different cross-head speeds.

Coherently, Figure 12 presents the thermal expansion recorded in the dogbone sample at the end of the deformation process (200 s) for three different cross-head speeds, as follows: $\dot{ʋ}$ = 1 mm/min, $\dot{ʋ}$ = 2 mm/min, and $\dot{ʋ}$ = 3 mm/min. The temperature distribution is illustrated using a color gradient, with corresponding maximum temperature values indicated for each strain rate.

At the lowest cross-head speed ($\dot{ʋ}$ = 1 mm/min), the temperature rise is minimal, as the slow deformation allows for better heat dissipation.

At moderate cross-head speed ($\dot{ʋ}$ = 2 mm/min), the temperature increases more, reflecting the increased energy input into the material.

The highest cross-head speed ($\dot{ʋ}$ = 3 mm/min) results in the maximum temperature observed, indicating that the heat generated by rapid deformation starts to exceed the rate at which it can be dissipated.

**Figure 13:**
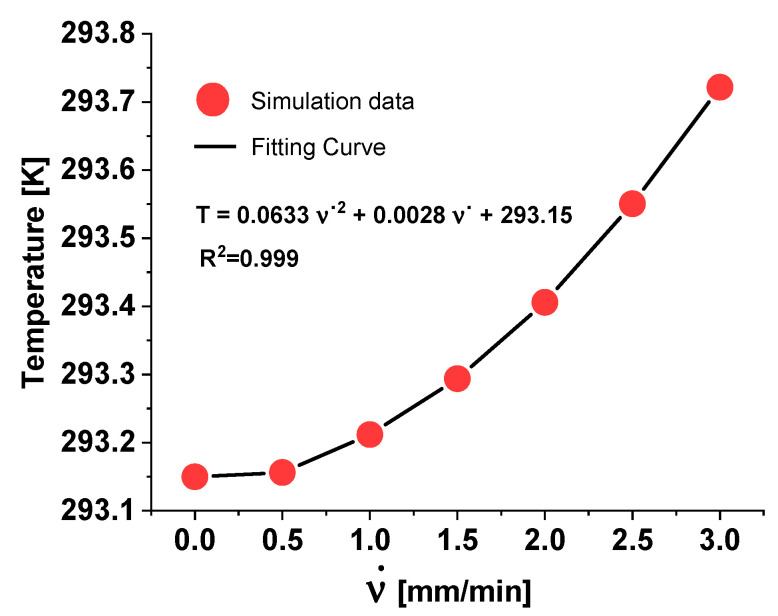
Temperature as a function of the cross-head speed.

By performing additional numerical simulations at further cross-head speeds (i.e.,$\dot{\;ʋ}$ = 0.5 mm/min and $\dot{ʋ}$ = 1.5 mm/min), it is possible to plot a graph of the strain-induced temperature variation with respect to the cross-head speed, as shown in Figure 13. A parabolic trend relates the two considered quantities.

**Figure 14:**
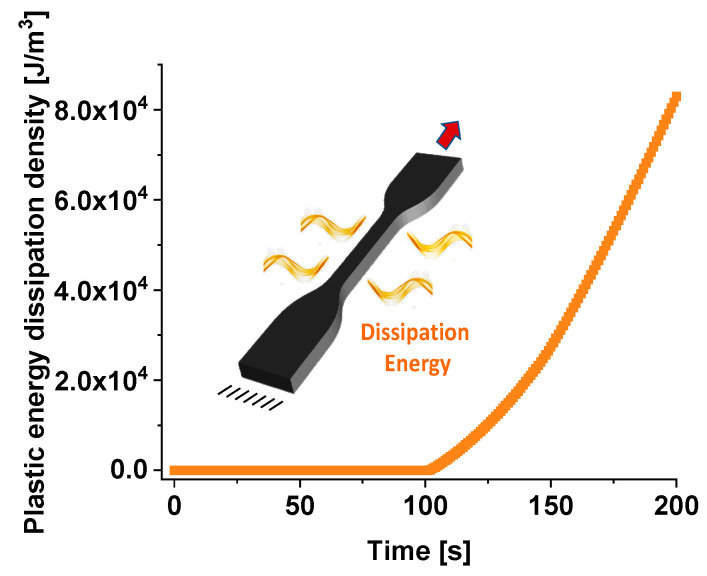
Plastic energy dissipation density (average value) over time.

Energy dissipation density typically refers to the distribution and rate of energy loss in a material due to plastic deformation and other internal processes during mechanical loading. In the context of composites under tensile stress, it encompasses the conversion of mechanical work into heat and other forms of internal energy dissipation. Figure 14 shows the plastic energy dissipation density (J/m^3^) over time (up to 200 s) evaluated on the total dogbone volume.

During the initial phase, from 0 to approximately 100 s, the plastic energy dissipation density is close to nonexistent. This suggests that there is minimal plastic deformation occurring within the specimen during this period; the composite material is undergoing elastic deformation initially, in which the energy is stored rather than dissipated as plastic deformation.

Exceeding 100 s, there is a noticeable increase in the plastic energy dissipation density. The curve begins to rise more steeply, indicating the onset of plastic deformation within the material. In more detail, as time progresses, the curve exhibits an exponential-like rise, reaching up to 8.0 × 10^4^ J/m^3^ at 200 s.

The stress distribution in the dogbone specimen is not uniform. The gauge region (central narrow part) typically experiences the highest stress concentration and thus the most significant plastic deformation. This leads to higher plastic energy dissipation densities in this region, in which the material dissipates energy through mechanisms such as dislocation movement, microstructural changes, and internal friction. The ends of the dogbone, where the specimen is clamped, experience different stress states. These areas might show lower energy dissipation density due to the constraints imposed by the fixtures and possible compressive stresses.

**Figure 15:**
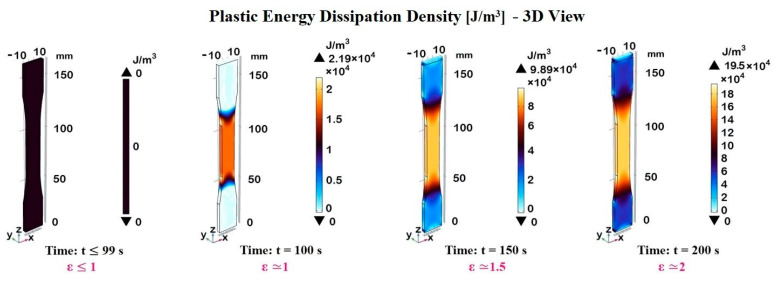
3D views of plastic energy dissipation density at different time instants/strain levels.

To appreciate this, Figure 15 presents 3D views of plastic energy dissipation density (measured in J/m^3^) evaluated at different time instants and strain levels during the deformation process. Each subfigure corresponds to a specific time and strain level, with color bars indicating the range and magnitude of the plastic energy dissipation density. For t ≤ 99 s corresponding to a strain of ε ≤ 1, the 3D model appears entirely black, indicating that there is no plastic energy dissipation density in this initial stage, or that it is negligible. During this phase, the material behaves elastically, meaning the deformation is reversible or, in other words, energy is stored in the material as elastic strain energy. As the time/load increases, the material enters the plastic region, where permanent deformation occurs.

At the time t = 100 s (ε ≈ 1) the 3D model shows areas of blue and orange colors. The blue regions represent lower values of energy dissipation density, while the orange regions represent higher values, up to 2.19 × 10^4^ J/m^3^.

This indicates the beginning of plastic deformation in specific areas. At the time that t = 150 s (ε ≈ 1.5), the 3D model has more pronounced blue and orange regions compared to the previous time instant. The orange regions are more extensive and intense, indicating an increase in plastic energy dissipation density, with a maximum value of 9.89 × 10^4^ J/m^3^. This suggests the progression of plastic deformation as time and strain increase.

Finally, at the time t = 200 s (ε ≈ 2), it is possible to note even more extensive areas of orange and blue. The maximum energy dissipation density has increased to 19.5 × 10^4^ J/m^3^. This indicates further progression and intensification of plastic deformation. Therefore, Figure 15 effectively illustrates the dynamic changes in plastic energy dissipation density during the deformation process, highlighting how both time and strain contribute to the evolving material response.

### 3.4. Thermo-Electric Properties: Experimental Results

**Figure 16:**
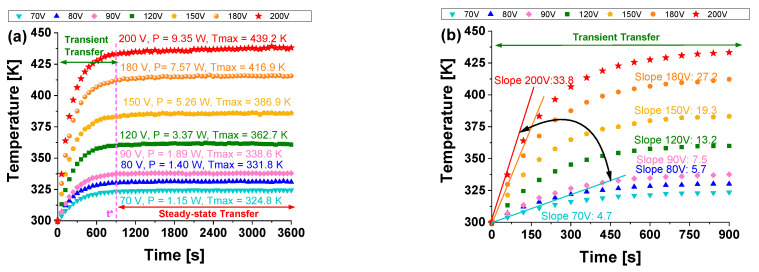
(**a**) Temperature patterns resulting from Joule heating at various applied voltage levels (from 70 V up to 90 V); (**b**) timeframe zoom of transient heat transfer for determining the heat rates for each voltage value.

Figure 16a shows the experimental variation of the top surface temperature over time (up to 3600 s) for nanocomposites, including 3 wt% of MWCNTs, subjected to diverse voltage levels (from a minimum of 70 V to a maximum of 200 V).

Physically, it is evident that heat transfer in solids starts as transient and then reaches a steady state, indicating a thermal equilibrium condition.

Specifically, during the transient interval, when the heat flow rate is changing, the temperature is time-dependent, T = T (t), for a brief period (t* marked in the figure). The temperature rises rapidly, then gradually approaches constant steady-state values of approximately 324.8 K and 439.2 K for 70 V and 200 V, respectively.

In the same figure, the corresponding electrical power dissipation (in watts) is shown for each experimental temperature curve. Increasing the voltage from 70 V to 200 V increases the power from 1.15 W to 9.35 W, respectively.

During steady-state transfer, which is marked by a consistent and specific heat transfer rate, the temperature remains constant over time.

This equilibrium occurs because the total heat generated by Joule heating is balanced by natural convection dissipating heat to the surrounding environment. Referring to Figure 16b, it is feasible to compute the heat rates (HR) as slopes of the respective temperature curves at the initial moments of the transient stage.

**Figure 17:**
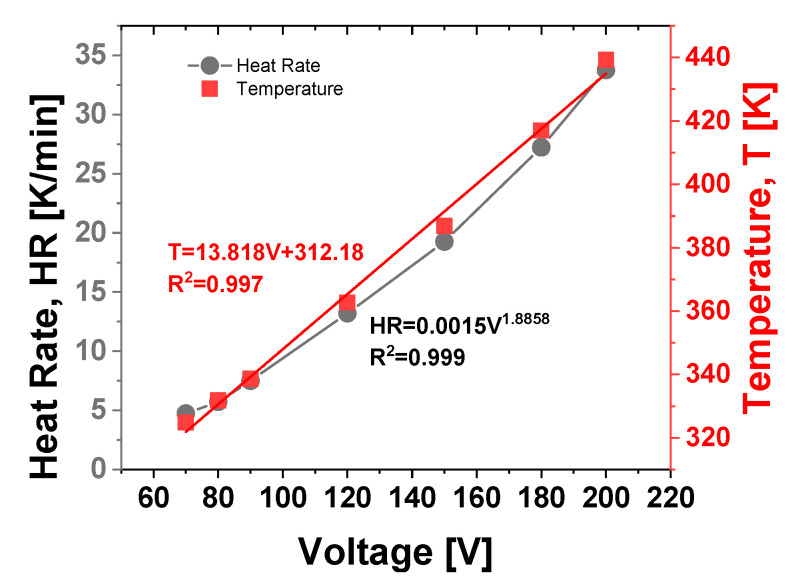
Experimental data and corresponding curve fitting of the temperature values recorded at t = 3600 s and heat rate as a function of the voltage levels in the right and left axes, respectively.

Specifically, as depicted in Figure 17 (right axis), these temperature values at steady-state (t = 3600 s) exhibit a linear relationship (with the coefficient of determination R^2^ approaching 1) with the voltage levels applied. This finding aligns with theoretical expectations, as the Joule effect, akin to thermal power dissipation P, varies linearly with voltage V, following the equation P = V⋅I, where I represents the electric current. Contrarily, as depicted in Figure 17 (left axis), an almost flawless exponential interpolation (alternatively, R^2^ = 0.973 or R^2^ = 0.981 is achieved with linear and power law fitting curves, respectively) is observed in the experimental data relating to the heat rate, HR [K/min], in relation to the applied voltage.

For quick referencing, these experimental thermal and electrical data depicted in their respective graphs are summarized in Table S2 of SM.

### 3.5. Design of Experiment (DoE) for a Selected Temperature during the Transient and Steady-State Phase

**Figure 18:**
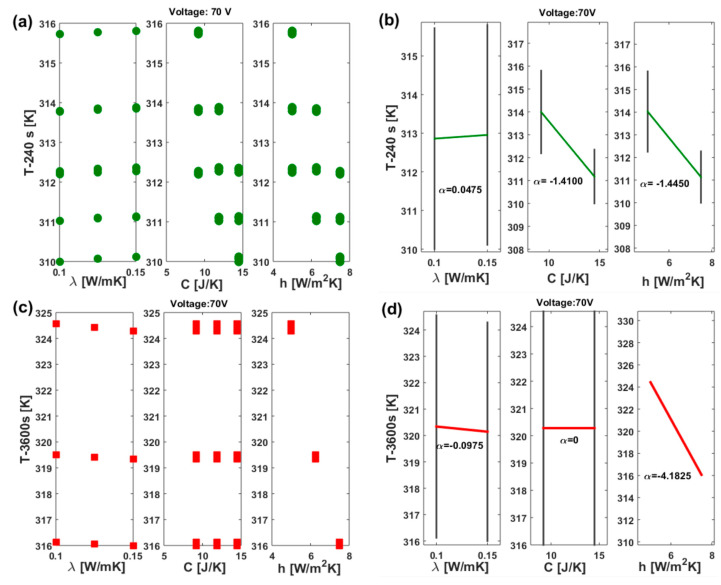
DSP and MfP for the upper surface temperature of the sample powered with 70 V. More specifically, (**a**) DSP at 240 s; (**b**) MfP at t = 240 s; (**c**) DSP at 3600 s; and (**d**) MfP at t = 3600 s.

**Figure 19:**
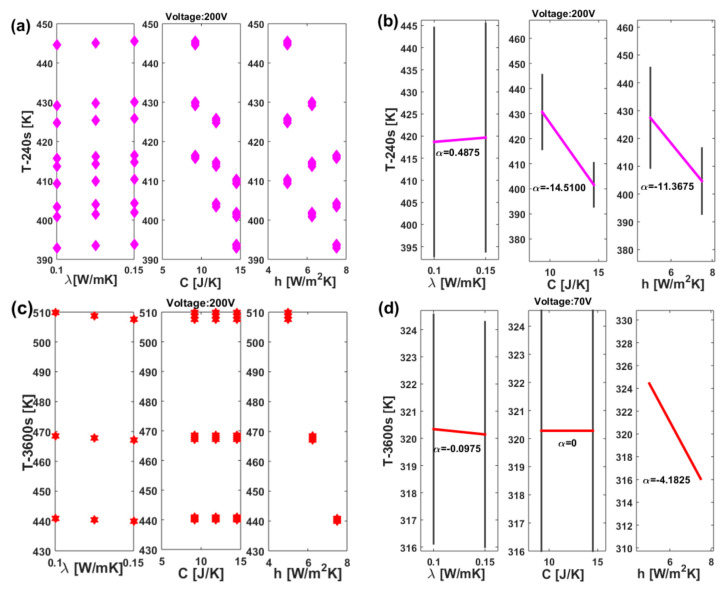
DSP and MfP for the upper surface temperature of the sample powered with 200 V. More specifically, (**a**) DSP at 240 s; (**b**) MfP at t = 240 s; (**c**) DSP at 3600 s; and (**d**) MfP at t = 3600 s.

The DoE method results in the dex scatter plot (DsP) and main factor plot (MfP) illustrated for nanocomposites subjected to 70 V and 200 V in Figure 18 and Figure 19, respectively. More specifically, in Figure 18 the DsP is shown in (a) and in (b) the corresponding MfP at time t = 240 s, while the related graphs evaluated at time t = 3600 s are displayed in (c) and (d), respectively. Similarly, in the case of DoE conducted with an applied voltage of 200 V, as shown in Figure 19, the DsP and associated MfP evaluated at time t = 240 s are presented in (a) and (b), respectively, whereas the corresponding results at time t = 3600 s are shown in (c) and (d).

In summary, a DSP chart displays the scattered data of the performance function (P.F.), such as the upper surface temperature at the instant of time t = 240 s and at t = 3600 s in our study (indicated as T-240s and T-3600s), on the vertical axis, against the independent variable displayed on the horizontal axis, i.e., the thermal conductivity (λ), the heat transfer coefficient (h), and the thermal capacity (C) in the current study. Graphically speaking, the DSP highlights how the P.F. responds to fluctuations of these independent factors [30,31]. Technically, their influence on the P.F. can be assessed by examining the MfP graphics and, in particular, the slope of the line connecting the average points of the performance function’s range values at the minimum and maximum levels of each factor. For a single factor, a horizontal line (parallel to the x-axis) signifies no effect on the P.F., while a sloped line indicates some level of influence, which can be measured and compared to the effects of other investigated factors [32].

According to these guidelines, it is possible to emphasize the distinct and joint effects of each thermal factor on the surface temperature due to Joule heating at the two selected time instants (240 s and 3600 s). Both the temperature values at t = 240 s, regardless of the applied voltage, are impacted by all three factors, albeit to varying entities and in different manners. An increase in thermal conductivity, within the range explored in this study, leads to a slight rise in the analyzed temperature values. This impact is quantifiable through the slopes highlighted by the MfP graphs, which amount to 0.0475 and 0.4875 for the respective applied voltages of 70 V and 200 V (see Figure 18b and Figure 19b).

On the other hand, an increase in both the heat transfer coefficient and thermal capacity results in a more significant reduction in the recorded temperature value at the time instant t = 240 s compared to the influence exerted by thermal conductivity. Specifically, these reductions are quantified at −1.4100 and −1.4450 for C and h when 70 V is applied. These values increase to −14.5100 and −11.3675, respectively, when 200 V is applied.

Referring to the equilibrium temperature values (t = 3600 s), the situation undergoes significant changes. Specifically, it is interesting to observe that the influence of thermal capacity becomes null (α = 0, see Figure 18d and Figure 19d), regardless of the voltage level. Conversely, the impact of thermal conductivity is reversed, since now its increase leads to a slight decrease in the maximum recorded temperature. This finding aligns with theoretical principles, in which higher thermal conductivity corresponds to a lower temperature gradient in a solid. Notably, the dominant effect of the heat transfer coefficient persists; in both scenarios (V = 70 V and V = 200 V), an increase significantly reduces (α = −4.1825 and α = −34.1475, respectively) the plateau temperature value. This outcome is consistent with theoretical expectations, as a higher heat transfer coefficient results in the heated sample remaining cooler by transferring more heat to its surrounding environment.

Table S3 of SM summarizes, for the two voltages under consideration, the estimated (with the statistical DoE approach) slope values indicating the influence of the three thermal parameters on the two temperatures of interest (t = 240 s and t = 3600 s).

### 3.6. Response Surface Methodology (RSM) for the Investigated Temperature Values

Response surface methodology (RSM), initially introduced by Box and Wilson [33] in the early 1950s, continues to be a widely used mathematical tool based on the design of experiments. It is employed to predict the relationship between multiple design parameters and experimental outcomes. Given the unknown form of the performance function (P.F.), the response surface methodology (RSM) aims to predict the response surface (R.S.), identifying optimal response regions amidst design input changes. The R.S. is typically expressed as follows:

(8) $$R.S.=f\;\left(X_{1},{\;X}_{2},\;\ldots X_{n}\right)+\varepsilon$$

where *f* is the mathematical relation between the *R.S.* and the independent input parameters (*X_i_*), and *ε* is the experimental error that follows a normal distribution with a mean of zero and constant variance. Polynomial models are commonly used for surface prediction; first-order (linear) or second-order (quadratic) models as used in the present study, are typically adequate for evaluating the performance of different problems in several contexts, particularly when it depends on two/three input parameters (e.g., thermal conductivity λ, thermal capacity *C*, and heat transfer coefficient *h* in our case) [34,35].

Mathematically speaking, the quadratic polynomial model (*n* = 3) can be described using the following equation:

(9) $$R.S.=\beta_{0}+\sum_{i=1}^{n}\beta_{i}x_{i}+\sum_{i=1}^{n}\beta_{ii}x_{i}^{2}+\sum_{i=1}^{n-1}\sum_{j=i+1}^{n}\beta_{ij}x_{i}x_{j}$$

where *x_i_* and *x_j_* represent the independent input parameters, and *β_0_* is the intercept coefficient, whereas *β_i_*, *β_ii_*, and *β_ij_* are the linear, quadratic, and interaction regression coefficients, respectively. These variables are evaluated using the least squares method. With reference to the temperature values T at 240 s and 3600 s (i.e., *T* − 240*s* and *T* − 3600*s*, respectively), the intention is to find an analytical function relating these selected temperature values with the thermal parameters taken into consideration in the current study, i.e.,$\;T-240s=f\left(\lambda ,C,h\right)$ = $f\left(x_{1},x_{2},\;x_{3}\right)$ for a more appropriate mathematical representation and $T-3600s=f\left(\lambda ,h\right)$ = $f\left(x_{1},\;x_{3}\right)$.

Based on equation (9), the quadratic polynomial approximating the dependent variable T is expressed as follows:

(10) $$\begin{array}{c} T-240s=f\left(x_{1},x_{2,}x_{3}\right)\\ \;\;\;\;\;\;=\beta_{0}+\beta_{1}x_{1}+\beta_{2}x_{2}+\beta_{3}x_{3}+\beta_{12}x_{1}x_{2}+\beta_{13}x_{1}x_{3}+\beta_{23}x_{2}x_{3}\\ \;\;\;\;\;\;+\beta_{11}x_{1}^{2}+\beta_{22}x_{2}^{2}+\beta_{33}x_{3}^{2} \end{array}$$

(11) $$T-3600s=f\left(x_{1},x_{3}\right)=\beta_{0}+\beta_{1}x_{1}+\beta_{3}x_{3}+\beta_{13}x_{1}x_{3}+\beta_{11}x_{1}^{2}+\beta_{33}x_{3}^{2}$$

The analysis is conducted with reference to the two extreme values of applied voltage, namely, 70 V and 200 V. All coefficients of the RSM are stated in Table 1.

**Figure 20:**
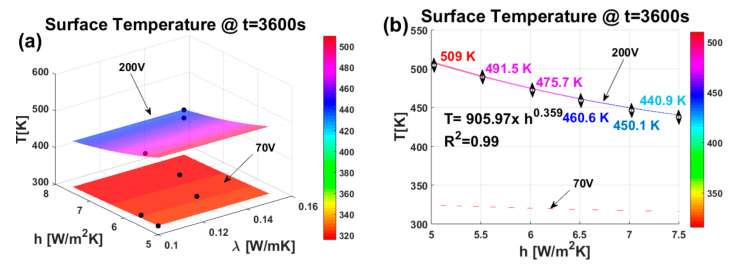
(**a**) 3D response surface graphics (full quadratic model) for the temperature, depending on thermal conductivity and heat exchange coefficient. The black markers represent the experimental thermal data; (**b**) 2D view of the interpolating surfaces as a function of the exclusive heat exchange coefficient.

Figure 20 shows, in (a), the 3D graphics of the two response surfaces at t = 3600 s for the two voltage levels selected for this investigation (70 V and 200 V) and, in (b), the exclusive 2D view of the surface temperature T as a function of the heat transfer coefficient (h).

Figure 20a demonstrates that the response surface (R.S.) closely matches the experimental data (indicated by black markers). This strong correlation confirms the accuracy of the regression model (RSM) in estimating physical properties and performance in relation to the conditioning parameters also used in the field of nanocomposites.

From the 2D view in Figure 20b, the influence of the heat transfer coefficient on the surface temperature can be better appreciated. While the curve corresponding to 70 V appears mostly flat due to the low heating of the solid by the Joule effect, the curve obtained with a voltage of 200 V shows a decrease following a power law (see equation and corresponding R^2^ in the figure) of the temperature as the *h* factor increases with the combined effect of the thermal conductivity variation.

This is due to the more efficient heat exchange between the solid and the surrounding environment that avoids the sample’s overheating.

**Figure 21:**
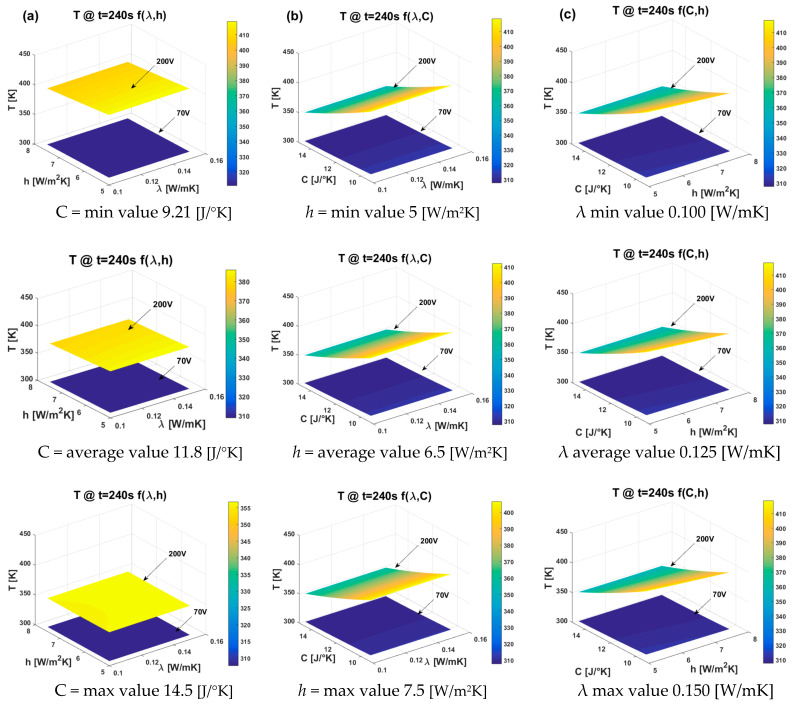
Response surface graphics presenting the effect of the three thermal factors on the surface temperature value recorded at the time instance of 240 s, as follows: (**a**) effect of thermal conductivity and heat transfer coefficient, (**b**) effect of thermal conductivity and thermal capacity, and (**c**) effect of heat transfer coefficient and thermal capacity. In each plot, the unconsidered factor was held constant at its minimum, average, and maximum values (first, second, and third row, respectively).

Figure 21 displays all the three-dimensional response surface plots for any pair of the three variable thermal factors.

Each plot features a vertical line (z-axis) representing the upper surface temperature [K] and two horizontal lines (x and y axes) showing the actual values of the selected explanatory factors within their respective ranges.

More in detail, Figure 21 shows, in (a), the effects of the thermal conductivity and heat transfer coefficient, in (b), the effects of the thermal conductivity and thermal capacity, and in (c), the effects of the heat transfer coefficient and thermal capacity. In each plot, the factor not represented on the horizontal axes was held constant at its minimum, average, and maximum values (first, second, and third row, respectively).

From the comparison of these plots, it is once again possible to confirm the analysis derived from the DoE. It is evident (with particular reference to the applied voltage of 200 V) that the thermal capacity *C* plays a dominant role in determining the temperature *T* reached on the sample’s surface during the transient heating phase (specifically at t = 240 s). Thermal conductivity has a negligible influence, whereas the contribution of the heat transfer coefficient *h* is appreciable (as seen from the different values of the corresponding color bars).

### 3.7. Thermo-Electric Properties: Simulation Results

**Figure 22:**
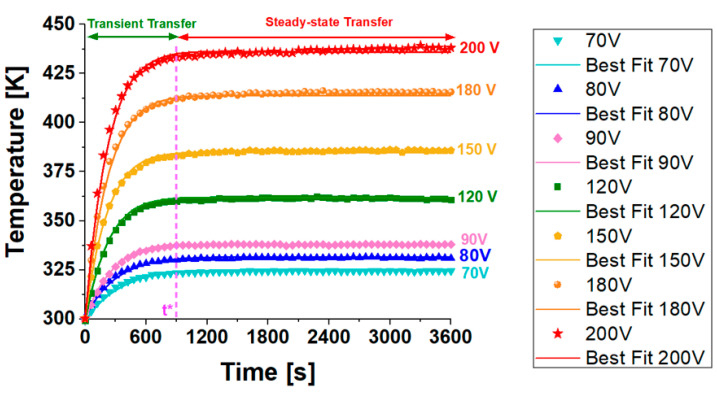
Simulation fitting for the temperature patterns for each applied voltage value.

Utilizing the DoE and RSM results, as well as the related considerations presented in the previous sections, it was possible to easily identify the thermal parameters λ, *C*, and *h* to be adopted in the various simulations. This ensured that the experimental curves regarding the surface temperature evolution of the sample at different applied voltage values could be accurately reproduced (see Figure 22) in the simulation environment of COMSOL Multiphysics used in this study.

It is worth noting that for each applied voltage level, the experimental and simulated curves of the sample’s surface temperature perfectly overlap in the transient and steady-state phases. Table 2 summarizes the values of the thermal parameters used in the simulations that fit the experimental data for the different voltage levels.

#### 3.7.1. Joule Heating Effect: Simulation Results

**Figure 23:**
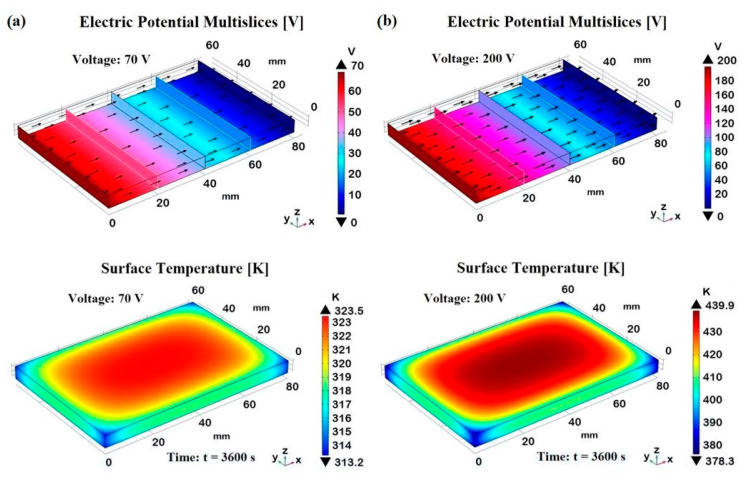
Electric potential distributions (first row) along the x-axis direction to which the two voltages (70 V and 200 V) in (**a**,**b**), respectively, are applied. The second row reports the 3D views of the corresponding surface temperature (evaluated at steady-state condition, t = 3600 s) due to the Joule effect.

Figure 23 illustrates the effects of different electric potentials on the temperature distribution due to the Joule heating effect. It includes two subfigures, (a) and (b), each representing a different applied voltage (70 V and 200 V, respectively), and shows both the electric potential distribution and the corresponding temperature distribution in the first and second row, correspondingly. The 3D plot shows a linear gradient in the electric potential from one end of the material (0 mm) to the other (80 mm), indicating a steady increase in voltage, from 0 V to 70 V in one case and from 0 V to 200 V in the other, across the length in the x-direction. The particular sectional views allow us to verify that, as expected, the electric potential assumes constant values in each transverse cross-section.

The black arrows indicate the current density, which, given the constant area, is greater for the sample subjected to a voltage of 200 V than for the one powered at 70 V.

The Joule heating is evident as the electric potential applied to the material generates heat, causing a temperature rise. The amount of heat generated is proportional to the square of the applied voltage (P = V^2^/R, where R is the electric resistance of the sample in Ohm), which explains the higher temperatures observed at 200 V (T ≃ 440 K at the time t = 3600 s) compared to that recorded at 70 V (≃ 324 K at the same considered time). Regardless of the voltage, the temperature distribution plots show a surface temperature increase, with the central region reaching the highest temperature, whereas it is noticeably lower near the borders and edges, highlighting significant heat dissipation due to natural convection with the surrounding air.

**Figure 24:**
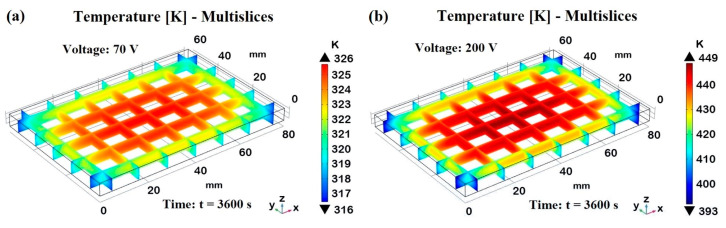
Multislice temperature distribution (evaluated at steady-state condition, t = 3600 s) due to the Joule effect for sample powered with 70 V in (**a**) and 200 V in (**b**).

This aspect is better investigated in Figure 24, which illustrates the multislice temperature distribution in the analyzed sample subjected to the Joule heating effect at the two different voltages, 70 V and 200 V in (a) and (b), respectively, both evaluated at steady-state conditions (t = 3600 s). The multislice temperature plots show how the temperature varies within the sample, emphasizing the central and edge regions.

In both cases, the central region of the samples reaches the maximum temperature of approximately 323 K and 440 K for 70 V and 200 V, as indicated by the corresponding orange and red areas in the plots. These result from significant Joule heating, in which the electrical energy is converted into heat, causing the central part to heat up more than the edges. In fact, moving from the center toward the edges, there is a clear temperature gradient. The temperatures decrease from the central orange/red areas to the blue areas near the edges, which are around 314 K and 378 K for the sample subjected to 70 V and 200 V, respectively. This gradient indicates effective heat dissipation at the boundaries due to natural convection with the surrounding air. In brief, both voltage levels show a central region with the highest temperatures and lower temperatures toward the edges, highlighting the effect of natural convection in cooling the sample periphery. Natural convection plays a crucial role here, as it removes heat from the sample edges more efficiently, preventing the outer regions from reaching the same high temperatures as the center. Higher voltages result in higher central temperatures and more pronounced gradients toward the edges.

#### 3.7.2. Spatial Temperature Distribution Simulation Results

Exploring the spatial temperature distribution in a solid due to Joule heating is essential for understanding how temperature varies throughout the solid and for predicting the material’s performance under operational conditions. Excessive localized heating can lead to thermal stresses, deformation, or even material failure.

**Figure 25:**
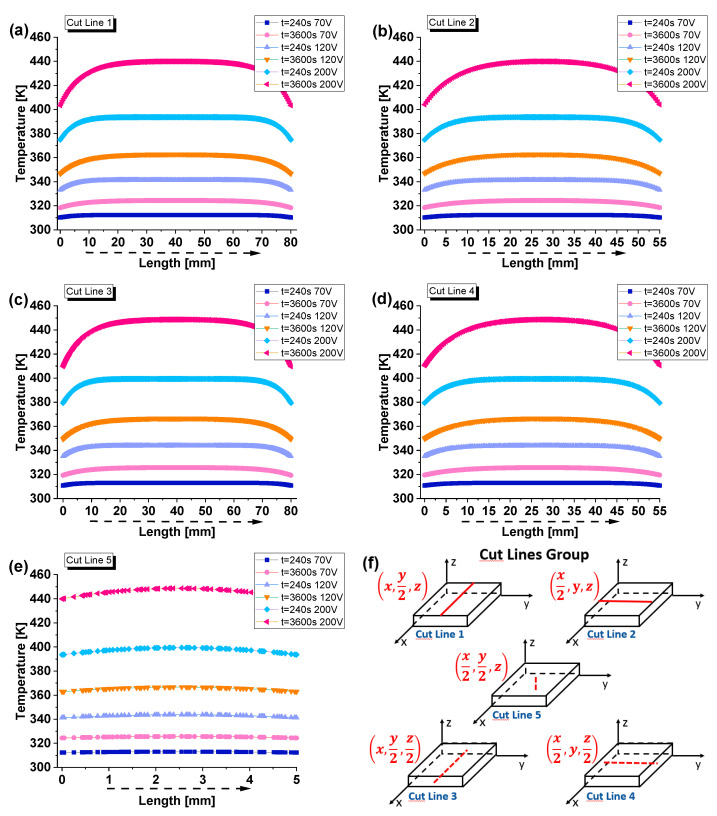
Temperature distribution along different spatial profiles, as follows: (**a**) cut line 1; (**b**) cut line 2; (**c**) cut line 3; (**d**) cut line 4; and (**e**) cut line 5. The cut line collection is schematized in (**f**).

Figure 25 shows the temperature distribution along different spatial profiles. In more detail, each subfigure from (a) to (e) shows the temperature distribution along a specific cutline at two different time points (t = 240 s and t = 3600 s) for voltage values 70 V, 120 V, and 200 V.

The cutlines are represented in subfigure (f) as follows:

- Cut line 1: parallel to the x-axis, on the top surface, at the midpoint of the sample’s width;
- Cut line 2: parallel to the y-axis, on the top surface, at the midpoint of the sample’s length;
- Cut line 3: parallel to the x-axis, on the median surface, at the midpoint of the sample’s thickness;
- Cutline 4: parallel to the y-axis, on the median surface, at the midpoint of the sample’s thickness;
- Cut line 5: parallel to the z-axis, at the midpoint of the sample’s width and length

(passing through the centroid of the sample along its thickness).

By analyzing these plots, it is possible to observe that for the sample powered with 70 V at t = 240 s and t = 3600 s, the temperature profiles are the lowest across all cut lines. The temperatures gradually increase along each dimension of the sample until they reach a maximum in the central area and then decrease again. Concerning 120 V, the temperature profiles show moderate heating, which becomes significant in the case of 200 V. At t = 3600 s, the temperature stabilizes at the highest values, reaching about 440 K. By comparing the results for cut lines 1 and 2 with those for cut lines 3 and 4, which differ only in their position along the thickness, it is worth noting that, although the trends are similar, the numerical values differ due to the varying Joule heating and the role played by convective heat exchange with the external environment. In particular, cut lines 3 and 4, located in the midpoint of the thickness, exhibit higher temperature profiles than cut lines 1 and 2, indicating a stronger effect of Joule heating at these positions. The heat remains trapped inside the solid, only partially reaching the external surfaces, from which it is then dissipated by convection with the external environment. For example, in the sample subjected to 200 V, the temperature recorded on the surface is approximately 440 K, while at the midpoint of its thickness, it reaches a value of about 448 K. This particularity is more clearly evident when referring to cut line 5, which allows for exploring the vertical temperature distribution along the z-axis. The trend shows higher temperatures at higher voltages, with significant differences between the initial and steady-state times. Notably, the maximum temperature value is recorded at the midpoint of the thickness (at the centroid). The lack of convective heat transfer on the solid’s inner surfaces prevents effective heat dissipation, leading to the accumulation of thermal energy in the central region. The heat generated by the Joule effect, following the passage of electric current under the effect of a potential difference, is not efficiently transferred to the external surfaces due to the relatively low thermal conductivity. As a result, the temperature rises significantly in this area, reaching its peak at the midpoint of the thickness, where the material’s thermal resistance is greatest. This phenomenon underscores the critical role of thermal conductivity, heat capacity, and convective boundary conditions in determining the temperature distribution within composite materials subjected to Joule heating.

#### 3.7.3. Convective Heat Flux: Simulation Results

**Figure 26:**
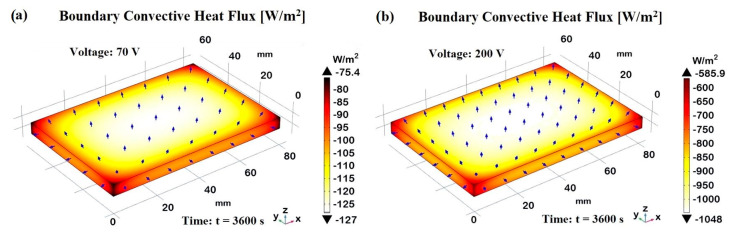
Comparison of the convective flux at t = 3600 s in case of applied voltage of 70 and 200 V in (**a**,**b**), respectively.

Effective thermal management is essential for the performance and longevity of devices designed for thermal applications. Convective heat flux, the transfer of heat from a surface to a surrounding fluid (usually air), plays a crucial role in dissipating heat and preventing overheating, and therefore, its investigation is essential. Figure 26 presents the boundary convective heat flux for the sample subjected to Joule heating at two extreme voltage levels, 70 V and 200 V, both evaluated at steady-state conditions (t = 3600 s), in (a) and (b), respectively. The color bars show the numerical values of the heat flux in W/m^2^, while the arrows indicate the magnitude and direction of the heat flux.

It is possible to note a relatively uniform distribution across the surface, but with a slightly higher concentration of arrows in the central region, indicating a more intense heat flux because of the greater exchange area compared to the other sides, which show less heat flux. At 70 V, the convective heat flux ranges from −75.4 W/m^2^ to −127 W/m^2^, indicating a moderate heat flux level across the sample. At 200 V, the convective heat flux ranges from −585.9 W/m^2^ to −1048 W/m^2^, showing a significantly higher heat flux level. This is due to the increased heating effect from the higher voltage, which results in more heat being dissipated through convection. The 3D views of the sample clearly demonstrate the impact of different voltages on the convective heat flux distribution on the samples subjected to Joule heating, as well as emphasize the role of natural convection in dissipating heat from the sample, especially from the central regions, where the heating is most intense.

## 4. Discussion

This study focuses on the experimental characterization, statistical, and numerical investigation of an epoxy resin composite loaded with varying concentrations of carbon nanotubes (CNTs) up to 3% by weight. These new nanocomposites, with enhanced mechanical, electrical, and thermal properties, are of significant interest for their potential use in practical applications for which, in the past, they were not taken into consideration due to their electric/thermal insulating nature. In actuality, carbon-based-reinforced epoxy resins represent a promising class of materials for advanced engineering applications, including thermal management aspects.

A preliminary characterization in terms of electrical conductivity as a function of filler concentration (percolation curve) allowed for the identification of two optimal concentrations for further investigation. Notably, the concentration of 0.5 wt% CNTs, corresponding to an electrical conductivity of 0.06 S/m, was selected for detailed studies on thermo-mechanical and piezoresistive properties. Concentrations near the electrical percolation threshold (EPT) are particularly suited for this type of analysis because the percolative network is just formed and, thus, more susceptible to deformations. It was experimentally observed that the sample exhibits elastic behavior up to strain levels of about 1% (yield point), beyond which it enters the plastic zone, leading to permanent deformations. This elastic–plastic transition is critical for understanding the mechanical properties of the nanocomposite. The slope of the linear region gives the Young’s modulus, indicating the material’s stiffness. Beyond the yield point, the material deforms plastically. Stress increases at a slower rate compared to the elastic region. The material undergoes significant permanent deformation in this region. The investigation of the variation in electrical resistance of the sample under applied strain (piezoresistive response) revealed that at the yield point, a distinct change in the slope of the resistance–strain curve was observed, confirming, once again, the transition from elastic to plastic behavior. This change is attributed to significant morphological alterations in the percolation network of CNTs under stress. The CNT network becomes disrupted in the plastic deformation region, leading to increased electrical resistance. This behavior underscores the sensitivity of the composite’s electrical properties to mechanical deformation, which is crucial for applications in sensors and structural health monitoring.

A numerical model based on the finite element method (FEM) using COMSOL Multiphysics^®^ (version 6.1) was adopted to predict the thermo-mechanical properties of the material by accurately reproducing (with CAD software) the dogbone-shaped sample, as required by measurement standards. As a first step, the proposed model was validated against experimental measurements (load as a function of strain).

The validation process involved comparing simulated load–strain curves with experimental data. This step ensured that the numerical model accurately represented the material behavior.

CNTs are known for their exceptional mechanical properties, including a high tensile strength and modulus. When dispersed in the epoxy matrix, they act as reinforcing agents, improving the overall mechanical performance of the composite. This effect is captured in the simulation. The good agreement between the experimental and numerical results is due to the careful choice of the values of the physical parameters, used to validate the simulation models, which are based on experimental data conducted in this and our previous studies. Parameters such as density, Young’s modulus, yield point, and other physical properties of the formulation containing 0.5 wt% of CNTs were accurately provided to the simulation software, leading to results that closely match the experimental data. Upon validation, the model was used to investigate the z-displacement in the direction of the applied force, providing insights into the deformation behavior under tensile loading. Both 3D visualizations and 2D plots were generated to illustrate these displacement behaviors of a dogbone composite material under prolonged loading. The increasing displacement over time and the significant strain within the gauge zone are key observations for understanding the material’s mechanical response and potential applications or limitations in structural contexts. The progressive increase in displacement indicated that the material does not immediately recover, which could indicate permanent deformation or damage accumulation over time.

Additionally, the distribution of Von Mises stress was analyzed to identify regions of high-stress concentration. The results indicated that the maximum stresses are concentrated in the central region of the dogbone sample, which is critical for understanding potential failure points. In the initial elastic region, as the material is strained, the stress increases linearly according to Hooke’s law, σ = Y⋅ε, where σ is the stress, Y is the Young’s modulus, and ε is the strain. This linear relationship means that as strain increases, the stress also increases proportionally, leading to higher Von Mises stress.

Contrarily, in the plastic deformation area, the material’s resistance to further deformation increases according to a physical phenomenon known as strain hardening. The Von Mises stress in the plastic region can be described using σ = σ_y_ + Y⋅(ε − ε_y_), where σ_y_ is the yield stress, ε is the total strain, and ε_y_ is the strain at yielding. This equation shows that the Von Mises stress increases as strain continues to accumulate beyond the yield point.

In brief, as the material deforms elastically and plastically over time, the internal resistance to deformation increases, necessitating higher stresses to continue the deformation process. This results in an overall increase in Von Mises stress, as numerically observed.

The thermal expansion of the sample under tensile testing was also examined. Heat dissipation during deformation may result from various mechanisms, such as internal friction between the material’s molecules, disordered movements of polymer chains, or plastic deformations involving sliding and dislocations in the material’s crystalline lattice.

In our case, the epoxy resin loaded with carbon nanotubes exhibited a low coefficient of thermal expansion (approximately 60 μ/K) and experienced minor plastic deformations (from 1% to 2% strain). Nonetheless, it was possible to graphically and quantitatively appreciate the overheating in the central region of the specimen. Although the recorded heating was not significant, this aspect should not be overlooked in other materials that experience more substantial plastic deformations

The effect of strain rate on strain-induced heating was also studied. Higher strain rates resulted in greater induced temperatures. This is because higher strain rates increase the rate of internal friction and mechanical energy dissipation, which converts more mechanical energy into heat. Understanding the thermal response of the material under different strain rates is crucial for applications in which the material is subjected to varying mechanical loads, as excessive heating could lead to material degradation or failure.

Finally, to complete this thermo-mechanical numerical study, the plastic energy dissipation was analyzed, which, as expected, only appears in the plastic zone and is not observed in the elastic zone. This is because plastic energy dissipation is associated with irreversible deformations, which do not occur in the elastic region. In brief, in the elastic region, mechanical energy is stored in the material as elastic strain energy, which can be fully recovered upon unloading. Consequently, there is no plastic dissipation in this region. However, in the plastic region, the material undergoes irreversible deformations, and the mechanical energy is dissipated as heat. This distinction was evident in the numerical simulations, in which plastic energy dissipation was observed only in the plastic zone. At the time t = 100 s (ε ≈ 1), the 3D model showed a plastic dissipation of about 2.19 × 10^4^ J/m^3^ that indicated the beginning of plastic deformation, whereas a maximum value of 9.89 × 10^4^ J/m^3^ is measured at t = 200 s (ε ≈ 2) thus suggesting the progression of plastic deformation over the time and strain increase.

Furthermore, as indicated in the title, this study also delved into the surface heating dynamics of composite materials driven by Joule heating at varying voltage levels. Joule heating, a fundamental process in which electrical energy dissipates as heat in the material, forms the cornerstone of our experimental investigation.

The self-heating capability of conductive nano-filled epoxy resins via the Joule effect for a variety of practical applications, such as self-repair, post-curing treatments of resins, and de-icing coatings was investigated by Prolongo et al., who found that composites filled with CNTs achieve higher temperatures than those enhanced with GNPs under low electrical voltage due to their superior electrical conductivity. Conversely, GNP/epoxy resins exhibit more uniform self-heating, owing to their greater thermal conductivity. It was also verified that the self-heating process is consistent across multiple cycles, attaining the same temperature when the same voltage is applied [36]. Such results are consistent with our previous study [37] and the main findings reported in the current work. Voltage levels ranging from 70 V to 200 V were systematically applied to evaluate their impact on surface temperature evolution. Beyond experimental observation, our approach integrated advanced design of experiments (DoE) techniques, employing dex scatter plot (DsP) and main factor plot (MfP) analyses. These methods allowed for the precise comprehension of how thermal parameters—thermal conductivity, thermal capacity, and heat transfer coefficient—affect temperature profiles during transient and steady-state heating phases. Through response surface methodology (RsM), we derived analytical relationships that explicitly correlate temperature variations with these thermal variables. Such statistical analyses revealed critical insights; within the studied voltage range, thermal conductivity exhibits minimal influence on temperatures during the heat transfer phase, such as 240 s, and during equilibrium (3600 s) plateau phases. Conversely, thermal capacity significantly affects temperatures during the initial heating phase but has a null impact on equilibrium temperatures. The heat transfer coefficient by natural convection h [W/m^2^K] emerges as a pivotal factor governing overall heating rates and plateau temperatures, adhering to the physical relationship known as Newton’s law of cooling, as follows: Q=h·S·∆T, where Q [W] is heat flow, and ΔT [K] is the temperature difference (T_Solid_ − T_room_) that gives rise to the convective transport between a heated solid surface S [m^2^] and the surrounding environment.

A further rigorous numerical model has been developed in COMSOL Multiphysics^®^. This model not only replicated experimental data accurately (this consistency served as its validation) but also facilitated a deeper exploration of thermo-electric properties, including the effects of natural convection at material edges, which induce localized cooling effects.

Future research aims to extend these insights by exploring modifications in heat sink geometries, such as edge smoothing, to optimize heat dissipation for cooling applications further or recover wasted heat for heating elements.

Therefore, insights gleaned from this integrated approach provide actionable strategies for optimizing heat exchanger designs and enhancing thermal management efficiency in various engineering applications.

In essence, this study establishes a robust framework for understanding and optimizing the thermal behavior of composite materials under mechanical stress and Joule heating, integrating advanced statistical analyses with validated numerical models to advance engineering design and innovation.

Creating new advanced hybrid materials using simulation studies and statistical techniques, such as DoE and RSM, can streamline the optimization process without requiring physical specimen testing. This approach cuts down on development time and the high costs of trial-and-error experiments. Additionally, the ideal composition and structure of new materials can be determined by integrating simulation methods with statistical tools. This combination also helps pinpoint the most influential controllable input factors, supporting design decisions during the manufacturing stage.

## 5. Conclusions

This study focused on the development and characterization of epoxy resins filled with varying concentrations of multi-walled carbon nanotubes (MWCNTs) up to 3 wt%. The experimental results highlighted the importance of the electrical percolation curve in identifying critical filler concentrations. Specifically, 0.5 wt% MWCNTs around the electrical percolation threshold were found suitable for exploring mechanical and piezoresistive properties. In contrast, a concentration of 3 wt% was optimal for investigating thermo-electric properties due to the Joule effect, with applied voltages ranging from 70 V to 200 V. Numerical models developed using Multiphysics simulation software were validated against experimental data, providing a robust framework for further investigation of the composites’ physical properties. Additionally, a statistical approach based on the design of experiments (DoE) was employed to understand the influence of thermal parameters on the final performance of the materials. Overall, the integration of experimental characterization with numerical modeling and statistical analysis has provided comprehensive insights into the behavior and potential use of MWCNT-epoxy composites in thermal management applications. This multidisciplinary approach not only enhances our understanding of these advanced materials but also paves the way for their optimized use in various industrial sections, particularly those in which enhanced thermo-mechanical and thermo-electric properties are crucial.

The conclusions drawn from this research support the broader goals of advancing materials science and engineering for improved performance and sustainability in various industries in several key ways. First of all, the research demonstrates that incorporating CNTs into epoxy resins significantly enhances their mechanical, electrical, and thermal properties. This improvement is crucial for developing high-performance materials that can meet the demanding requirements of advanced applications, such as thermal management systems. By identifying critical filler concentrations (0.5 wt% near the electrical percolation threshold (EPT) for thermo-mechanical properties and 3 wt% for thermo-electric properties), the research provides valuable insights into optimizing the concentration of CNTs to achieve desired material properties. This knowledge helps design materials with specific functionalities, such as deformation sensing and Joule heating, which are essential for various industrial applications. Moreover, the research emphasizes the use of contemporary statistical and computational techniques alongside experimental methods. The development and validation of numerical models using Multiphysics simulation software, combined with a statistical approach based on the design of experiments (DoE), provide a comprehensive understanding of the materials’ properties. This integrated approach enhances the accuracy and reliability of the research findings, facilitating the identification and optimization of new materials. Future research will continue to refine these models and explore additional parameters to optimize further the performance and applicability of these promising composite materials.

## Figures and Tables

**Table 1:** Coefficients for the quadratic response of the upper surface temperature, as determined usng RSM.

Coefficient	β_0_	β_1_	β_2_	β_3_	β_11_	β_22_	β_33_	β_12_	β_13_	β_23_
T-240s–70 V	+338.478	+5.560	−1.585	−3.368	−19.555	+0.018	+0.086	+0.100	−5.978 × 10^−13^	+0.097
T-3600s–70 V	+817.118	−91.299	0	−82.011	−2.666	0	+4.277	0	+9.839	0
T-240s–200 V	+640.033	+57.822	−16.136	−23.972	−174.222	+0.202	+0.321	+0.592	−0.320	+0.321
T-3600s–200 V	+362.323	−11.966	0	−10.068	+2.666	0	+0.5258	0	+1.199	0

**Table 2:** Thermal parameters for the simulation fitting.

	70 Volt	80 Volt	90 Volt	120 Volt	150 Volt	180 Volt	200 Volt
*C* [J/°K]	14.5	14.5	9.21	11.8	11.8	14.5	14.5
*λ* [W/mK]	0.100	0.100	0.100	0.125	0.125	0.125	0.150
*h* [W/m^2^K]	5	5.5	5	6	7	7.5	7.5

## Data Availability

Data are contained within the article.

## Supplementary Materials

The following supporting information can be downloaded at https://www.mdpi.com/article/10.3390/ma17143596/s1: Figure S1: Key features of the components of nanocomposites and test specimens; Table S1: Initial (I.C.) and boundary conditions (B.C.) for computing the eq. 1. Figure S2: Diagrammatic representation of a system (product or process) during the design phase; Table S2. Thermal and electrical experimental data determined for each voltage value; Table S3. Slope values for the thermal parameters *λ*, *C* and *h*.
