# Supplementary material for: Thermo-Mechanical and Thermo-Electric Properties of a Carbon-Based Epoxy Resin: An Experimental, Statistical, and Numerical Investigation
Source: Materials (Basel). 2024 Jul 21;17(14):3596. doi: 10.3390/ma17143596 (PMC11278644; doi:10.3390/ma17143596)
Supplement: Supplementary file 1 [file materials-17-03596-s001.zip › materials-3096287-supplementary.pdf]

Supplementary Material

Thermo-Mechanical and Thermo-Electric Properties of a Carbon-Based Epoxy Resin: An Experimental, Statistical, and Numerical Investigation

Giovanni Spinelli [1,2,*], Rosella Guarini [2,3], Liberata Guadagno [3], Luigi Vertuccio [4] and Vittorio Romano [3]

[1] Faculty of Transport Sciences and Technologies, Università Telematica Giustino Fortunato, Via Raffaele Delcogliano 12, 82100 Benevento, Italy
[2] Institute of Mechanics, Bulgarian Academy of Sciences, Open Laboratory on Experimental Micro and Nano Mechanics (OLEM), Acad. G. Bonchev Str. Block 4, 1113 Sofia, Bulgaria; rgrosagi@gmail.com
[3] Department of Industrial Engineering, University of Salerno, Via Giovanni Paolo II 132, 84084 Fisciano, Italy; lguadagno@unisa.it (L.G.); vittorioromano2022@gmail.com (V.R.)
[4] Department of Engineering, University of Campania "Luigi Vanvitelli", Via Roma 29, 81031 Aversa, Italy; luigi.vertuccio@unicampania.it
* Correspondence: g.spinelli1@unifortunato.eu

2. Materials and Methods

In the current investigation, the compounds used the precursor "3,4-Epoxycyclohexylmethyl3′,4′-epoxycyclohexane carboxylate" (ECC) and the hardening agent "Methyl hexahydrophthalic anhydride" (MHHPA). These components were supplied by Gurit (Gurit Holding Wattwil, Switzerland). The carbon nanotubes (GRAPHISTRENGTH C100), with a carbon purity exceeding 90% and weight loss observed at 105°C, were sourced from ARKEMA (ARKEMA Colombes, France).

For clarity and completeness, the following information is still provided. To achieve a homogeneous dispersion, the filler was incorporated into the precursor using ultrasonication for 20 minutes (Hielscher model UP200S-24 kHz high-power ultrasonic probe, Hielscher Ultrasonics, Teltow, Germany). A quantity of hardener was added to the filler/precursor mixture with a weight ratio of 1:1. The resulting mixture was stirred magnetically for 20 minutes at ambient temperature and subsequently degassed for 2 hours at room temperature. The prepared mixtures were cured using the following thermal cycle: 1 hour at 80 °C, 20 minutes at 120 °C, and 1 hour at 180 °C.

The essential physical and chemical characteristics of the precursor, hardener agent, filler, and the dimensions of the parallelepiped-shaped test specimens are concisely outlined in Fig. S1.

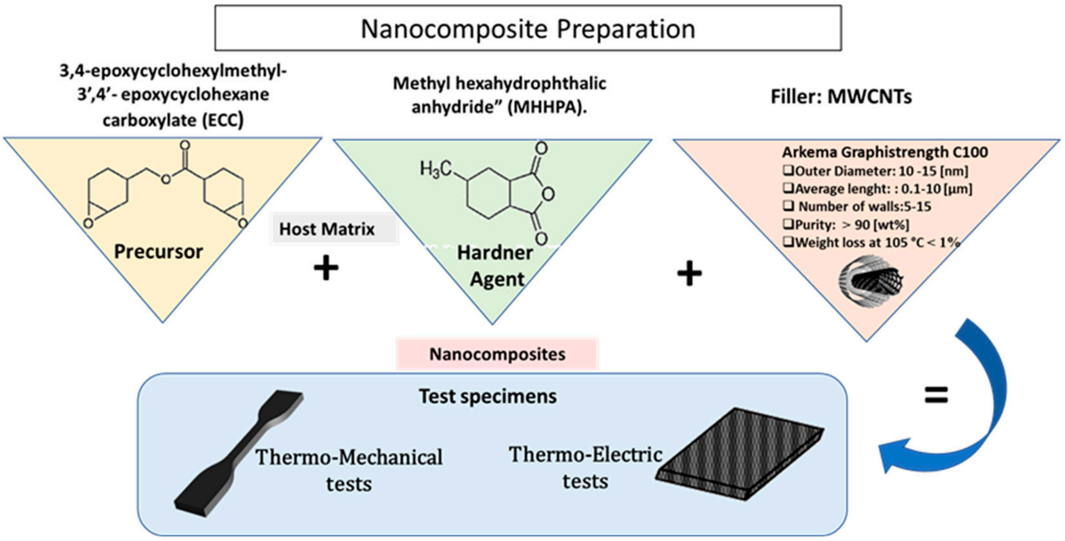

Figure S1. Key features of the components of nanocomposites and test specimens.

The physics module solid mechanics is used to simulate the mechanical behavior of the composite material under tensile loading. It helps in analyzing stress, strain, and deformation. Otherwise, the module heat transfer in solid state is used to model the heat generation and distribution within the material due to strain-induced heating and thermal expansion.

A coupled physics by combining Joule heating and heat transfer is adopted instead to numerically investigate the coupled thermo-electric behavior of the composite material. The physics module Joule heating is used to simulate the heating of an electrical nature due to the application of a current or voltage, whereas the module heat transfer in solid state is used to analyze the consequent thermal aspects that are triggered in the sample.

Referring to a differential volume ΔxΔyΔz, the equation (at constant pressure) governing heat transfer in a solid utilized in the simulations can be formulated in Cartesian coordinates as follows:

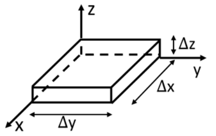

$$\frac{\partial}{\partial x}\left(\lambda\frac{\partial T}{\partial x}\right)+\frac{\partial}{\partial y}\left(\lambda\frac{\partial T}{\partial y}\right)+\frac{\partial}{\partial z}\left(\lambda\frac{\partial T}{\partial z}\right)+\begin{cases}Q|_{\text{Strain heating}}\\ Q|_{\text{Joule heating}}\end{cases}=\rho c_p\frac{\partial T}{\partial t} \qquad \text{(S1)}$$

where λ represents the thermal conductivity [W/mK], ρ is the density [kg/m^3] and c_p is the specific heat [J/kgK] of the materials. The term $Q_{Joule\ heating} = J\cdot E$ is the electrical power [W/m^3] generated by the electrical current for the Joule effect in the parallelepiped volume. In particular, J denotes the current density [Am^{-2}] equal to current (I) divided by the effective cross-section area (A) of the sample and E is the electric field strength [W/Am] caused by several voltage values (70V, 80V, 90V, 120V, 150V and 200V).
Similarly, $Q_{Strain\ heating} = \eta\cdot\sigma\cdot\dot{\varepsilon}_{plastic}$ is the power per unit volume of the dogbone sample [W/m^3], generated by plastic deformation that can be expressed as the product of stress (σ, [N/m^2]) and the rate of plastic strain ($\dot{\varepsilon}_{plastic}$,[1/s]). The term η (dimensionless) is the so-called mechanical dissipation factor or in other terms the fraction of mechanical energy converted into heat.

Table S1 compiles the initial and boundary criteria necessary for accurately resolving the thermal equilibrium described by equation (1) for both parallelepiped and dogbone geometry.

Table S1. Initial (I.C.) and boundary conditions (B.C.) for computing the eq. 1.

I.C. and B.C. conditions		Equations	Applicability
I. C.	t=0	T=Room Temperature (T_0)	$\forall x, \forall y, \forall z$
B. C.	*Top and Down Surfaces*	$-\lambda \frac{\partial T}{\partial z} = h \cdot (T - T_\infty)$	$(\forall x, \forall y, t > 0)$
B. C.	*Lateral Surfaces*	$-\lambda \frac{\partial T}{\partial y} = h \cdot (T - T_\infty)$	$(\forall x, \forall z, t > 0)$
B. C.	*Front and Back Surfaces*	$-\lambda \frac{\partial T}{\partial x} = h \cdot (T - T_\infty)$	$(\forall y, \forall z, t > 0)$

Additionally, in the numerical analysis, an electrical conductivity of $6.8 \cdot 10^{-2}$ [S/m] and a density of 1180 [kg/m^3] were utilized based on our experimental findings, while thermal conductivity, heat transfer coefficient, and heat capacity were adopted in alignment with the results obtained from the DoE study.

In summary, as illustrated in Fig. S2, controllable variables (Xi) and uncontrollable ones (Ni) can be considered during the design stage, which often includes noise and tolerances on Xi. The collective influence of these controllable ($X = (X_1, X_2, ..., X_P)$) and noise ($N = (N_1, N_2, ..., N_q)$) variables impacts the performance function of interest (P.F. = f(X, N)). Through the Design of Experiments (DoE), the most influential variable on the chosen P.F. can be identified, and the controllable input variables can be appropriately selected to optimize the performance. Consequently, optimal parameter settings can be determined to enhance the desired performance.

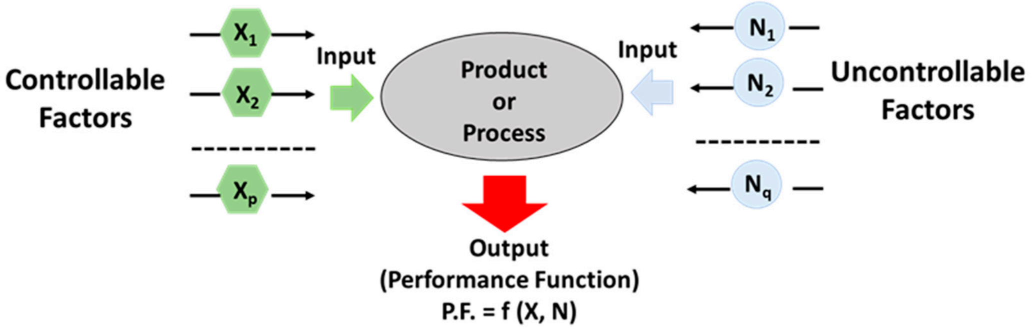

Figure S2. Diagrammatic representation of a system (product or process) during the design phase.

3. Results

3.4. Thermo-electric properties: experimental results

Table S2. Thermal and electrical experimental data determined for each voltage value.

	70 Volt	80 Volt	90 Volt	120 Volt	150 Volt	180 Volt	200 Volt
**Power [W]**	1.15	1.40	1.89	3.37	5.26	7.57	9.35
**T-3600s [K]**	324.8	331.8	338.6	362.7	386.9	416.9	439.2
**HR [K/min]**	4.7	5.7	7.5	13.2	19.3	27.2	33.8

3.5. Design of experiment (DoE) for a selected temperature during the transient and steady-state phase

Table S3. Slope values for the thermal parameters λ, C and h.

	**λ**	**C**	**h**
**α (T-240 s 70V)**	0.0475	-1.4100	-1.4450
**α (T-3600 s 70V)**	-0.0975	0	-4.1825
**α (T-240 s 200V)**	0.4875	-14.5100	-11.3675
**α (T-3600 s 200V)**	-0.7875	0	-34.1475
